# Single-crystal chiral two-dimensional supramolecular organic frameworks for tunable circularly polarized luminescence†

**DOI:** 10.1039/d4sc08811e

**Published:** 2025-03-19

**Authors:** Jialin Cui, Hui Wang, Hui Liu, Hailong Yu, Wei Wang, Yu Wang, Yingjie Zhao

**Affiliations:** a Qingdao University of Science and Technology 53 Zhengzhou Road Qingdao 266000 China hliu@qust.edu.cn yz@qust.edu.cn; b Shanghai Key Laboratory of Green Chemistry and Chemical Processes, School of Chemistry and Molecular Engineering, East China Normal University 3663N. Zhongshan Road Shanghai 200062 China yuwang@chem.ecnu.edu.cn

## Abstract

Chiral supramolecular organic frameworks (SOFs) and hydrogen-bonded organic frameworks (HOFs) remain an unexplored field, with very few reported examples. Here, three chiral SOFs with perfect two-dimensional (2D) framework structures are constructed by self-assembly between the chiral macrocyclic host molecule and different guest molecules through host–guest and hydrogen-bonding interactions. Variations in the guest structures lead to different host–guest interactions. The formation of the 2D frameworks of the chiral host and the guest molecules realizes chirality transfer and enhances the performance of circularly polarized luminescence (CPL) through strong charge transfer (CT) mechanisms, leading to the successful regulation of the CPL of the obtained SOF series. Chiral SOFs are significant enough due to their ability to combine chirality with versatile porous frameworks, leading to innovative solutions in optical devices, separations, catalysis, and beyond. Their tunability and eco-friendly synthesis further enhance their importance in chiral materials.

## Abstract 2

Chiral two-dimensional supramolecular organic frameworks (SOFs) with tunable circularly polarized luminescence (CPL) are constructed through the host–guest interactions between a chiral host and luminescent guests.

## Graphical abstract

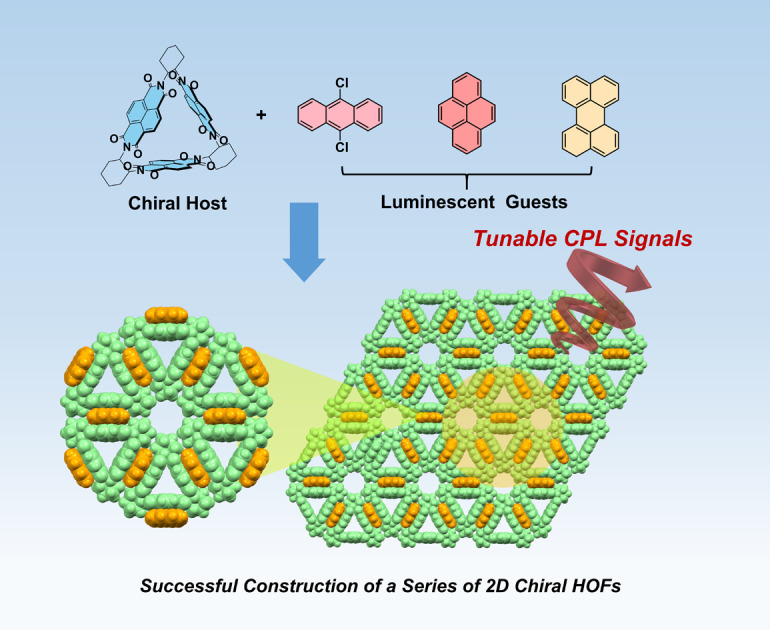

## Introduction

Supramolecular organic frameworks (SOFs) and hydrogen-bonded organic frameworks (HOFs) are a fascinating class of porous materials that are constructed through the self-assembly of organic molecules *via* hydrogen bonds and non-covalent interactions.^1–7^ Unlike metal–organic frameworks (MOFs) or covalent organic frameworks (COFs), SOFs and HOFs rely on weaker, reversible forces such as hydrogen bonding, π–π stacking, van der Waals forces, *etc.*^8–11^ Modularity is a notable characteristic of these materials, which endows them with design flexibility.^12–16^ The structure and properties of SOFs and HOFs can be easily adjusted by modifying the organic components and the conditions under which the framework is assembled. This tunability at the molecular level provides a high degree of control over the materials with specific functionalities tailored to particular applications. Another notable feature of these materials is the dynamic process of constructing these materials.^17–20^ The dynamic nature of non-covalent interactions makes the construction process of these materials relatively easy, and it is usually possible to obtain single-crystal samples. Through X-ray single-crystal diffraction, structural information at atomic-level resolution can be obtained. This allows us to gain a deeper understanding of structure–property relationships at the atomic level, enabling us to modify structures based on their performance.

Based on the aforementioned modularity and dynamic characteristics of SOF and HOF materials, it is reasonable to consider introducing chiral building blocks into the framework structure to construct chiral materials. Despite this, to our surprise, examples of chiral SOF and HOF materials are rare and precious.^7,21–24^ Designing chiral building blocks that can self-assemble into stable and well-defined frameworks is complex. The chiral building blocks must possess specific stereochemistry to induce and maintain chirality in the final framework structure. Chen and co-workers carried out the pioneering work on the construction of chiral HOF materials through chiral building blocks (BINOL).^7^ In addition, excellent examples have been reported in the construction of chiral supramolecular assembly^25–28^ and SOF materials through chiral building blocks.^29–32^ Nevertheless, the construction of chiral HOF or SOF materials still faces significant challenges.

As we know, chirality is a key concept that bridges multiple disciplines, influencing the development of new optical materials, the understanding of biological processes, and the advancement of asymmetric synthesis.^33–35^ Circularly polarized luminescence (CPL) active materials, as an important class of optical materials, hold significant importance in the application of three-dimensional (3D) displays and imaging, optical communication, chiral sensing, quantum cryptography, *etc.*^36–39^ SOFs offer significant advantages in the development of CPL materials due to their highly ordered structures with good structural tunability, which bring about versatile chemical functionalities. Leveraging the advantages of the tunable structures in SOF materials and the versatile host–guest chemistry as tools, the CPL signals in various SOFs can be modulated and amplified. Chiral supramolecular assemblies have demonstrated a remarkable ability to significantly amplify the circular polarization degree of CPL-active materials.^40–42^ In addition, due to the highly ordered structures, the aggregation-caused quenching (ACQ) effect of luminophores can be suppressed.^43,44^ This allows for highly efficient solid-state CPL-active SOF materials.^45^ Nevertheless, the examples of CPL-active materials based on SOFs are very limited, indicating that this field remains to be explored in depth. It is clear that the greatest challenge in this field lies in constructing high-quality CPL host–guest assembly frameworks that exhibit optimal chiroptical activity.

Here, a chiral macrocyclic host molecule was utilized for constructing two-dimensional (2D) chiral SOF single crystals with different guest molecules through hydrogen-bonding interactions between the 2D layers and host–guest interactions in the 2D layers. By adjusting the structures of guest molecules, the host–guest interactions in the frameworks can be adjusted, which further modulates electron coupling between the host and the guest, facilitating the regulation of CPL through host–guest assembly systems. Notably, due to the strong hydrogen-bonding interactions among the 2D layers and π–π interactions between the host and guest molecules in a single layer, the overall 2D framework structure is maintained despite changes in the structure of the guest molecules. In addition, the host–guest interactions are crucial for chiral transfer, playing a significant role in the overall chiral transfer (CT) process. These chiral SOF materials are interesting enough to validate and surpass some basic theories and provide precise models to comprehend the process of chiral transformation.

## Results and discussion

**Fig. 1:**
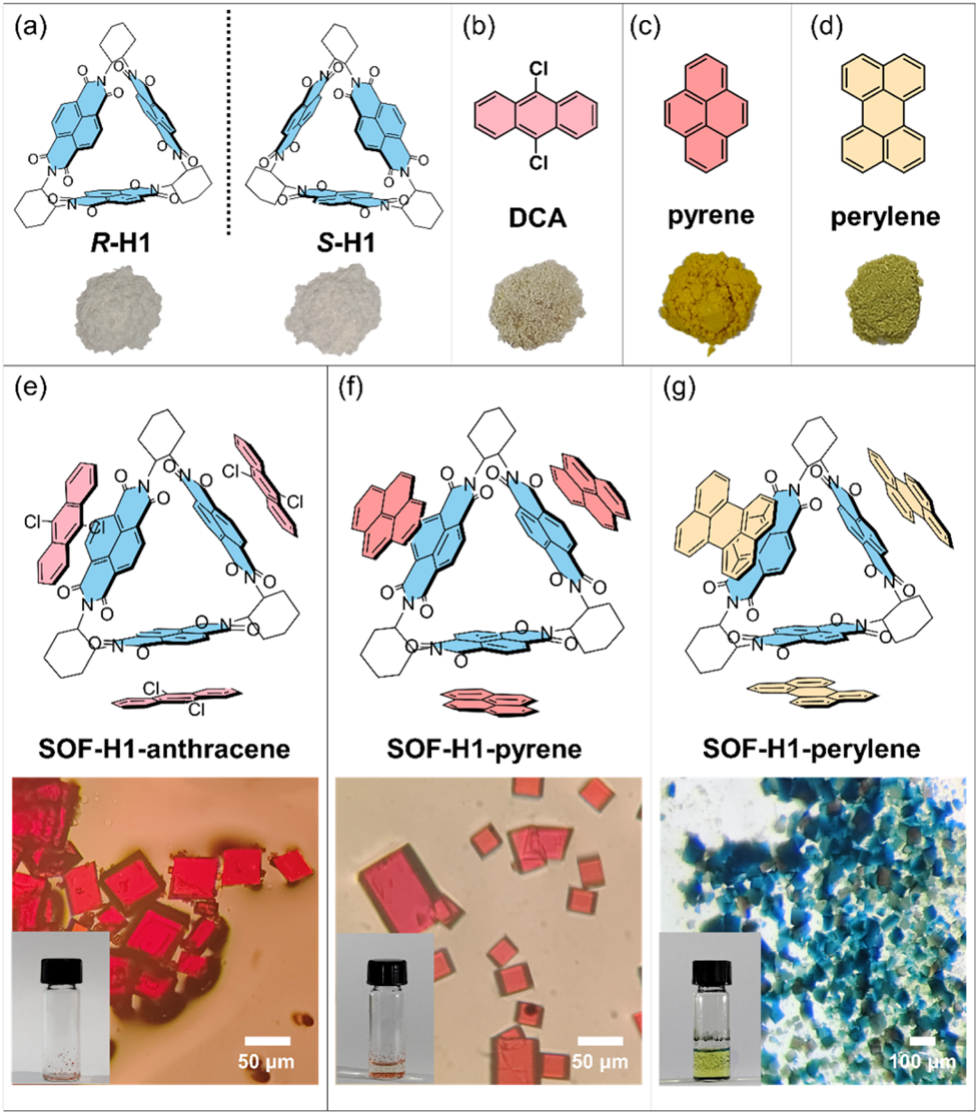
(a) The chemical structures of the enantiomers of H1 and their photographs. (b–d) The chemical structures of the guest molecules and their photographs. (e–g) The chemical structures of the three SOFs (SOF-H1-anthracene, SOF-H1-pyrene, and SOF-H1-perylene) and their optical microscope images and photographs. The *R* enantiomers were selected as representatives to present.

The synthesis of the chiral macrocyclic host molecule is very straightforward.^46–48^ The introduction of chirality relies on chiral cyclohexanediamine as the reactant. By simply changing the chirality of cyclohexanediamine, enantiomeric triangular macrocycles (*R*-H1 or *S*-H1) can be obtained (Fig. 1a). In the triangular macrocyclic host molecule, the moiety of naphthalene diimide (NDI) has been widely recognized as an electron-deficient π-system due to the presence of four electron-withdrawing carbonyl groups.^49,50^ Therefore, the macrocyclic host molecule can be considered to have three electron-deficient planes uniformly oriented in three directions within the 2D plane. Thus, by introducing appropriately sized planar electron-rich π molecules, self-assembly should occur in a 2D plane through donor–acceptor (D–A) interactions between the host and guest molecules, forming 2D framework structures.^51–53^ A series of π-conjugated planar molecules, including 9,10-dichloroanthracene (DCA), pyrene, and perylene were chosen as the guest molecules (Fig. 1b–d). With the π-conjugated systems gradually increasing in size, they exhibit well-defined regularity with enhanced electron donating behavior.

**Fig. 2:**
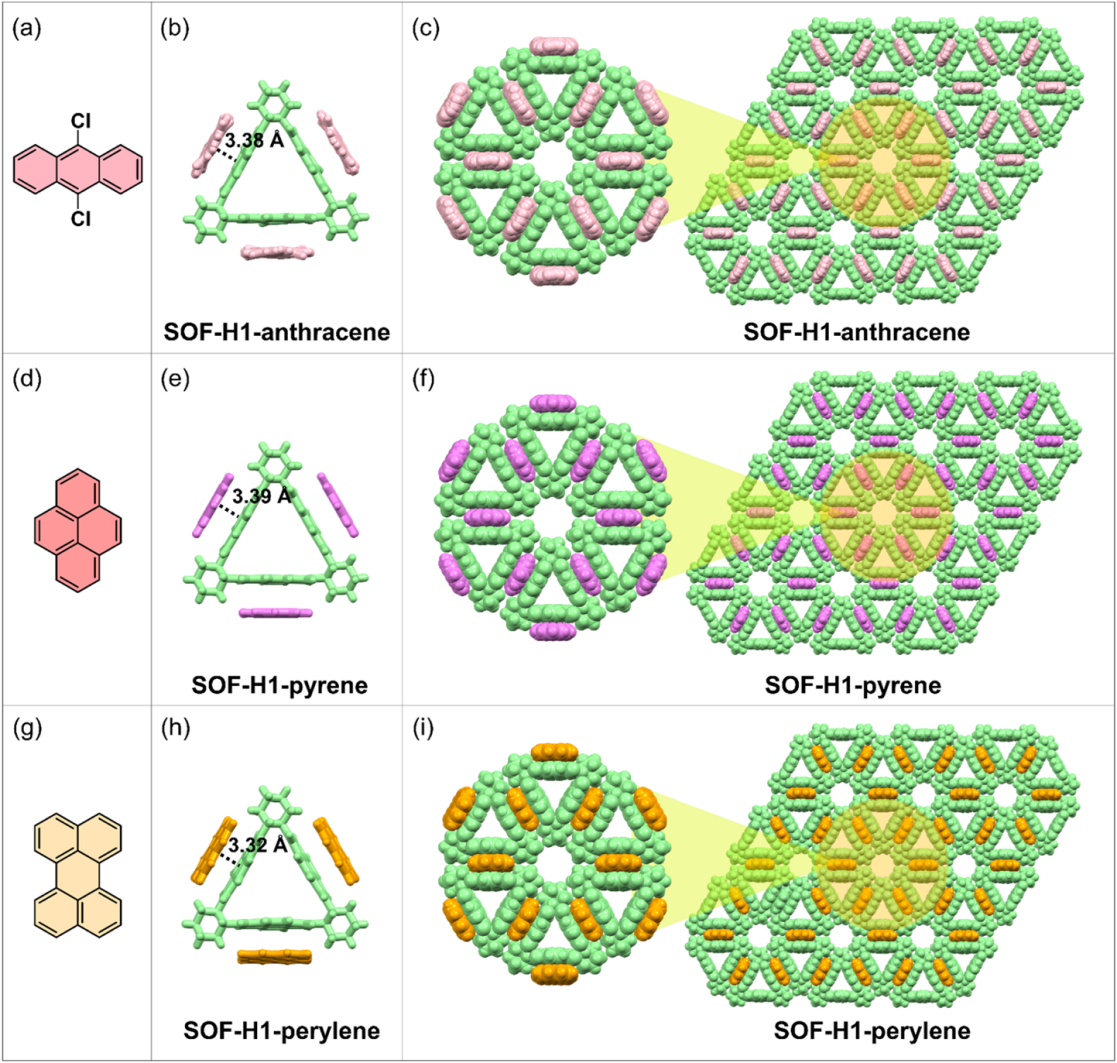
(a, d and g) The chemical structures of the three guest molecules. (b, e and h) The single-crystal structures of the SOFs showing the π–π interactions between the host and guest molecules. (c, f and i) The single-crystal 2D framework structures of the three obtained SOFs in the *ab* plane along the *c*-axis. The *R* enantiomers were selected as representatives.

Fortunately, the single crystal of the host macrocyclic molecule itself and the host–guest co-crystal can be easily grown in common solvents through the two-phase diffusion method or slow solvent evaporation. The host molecule H1, serving as the acceptor molecule, appears as a white powder, while the selected electron donors DCA, pyrene, and perylene are yellow, white, and bright yellow powders, respectively (Fig. 1a–d). The co-crystal of H1 and DCA has been reported by Stoddart and co-workers.^53^ Dissolving white H1 and yellow DCA in dichloromethane forms a colorless solution, but as methanol vapor slowly diffuses in, a large number of red rectangular SOF-H1-anthracene co-crystals were obtained (Fig. 1e). Dichloromethane solvent was gradually added to the mixture of pyrene and H1. The solution quickly turns red and then becomes colorless. The red quadrilateral SOF-H1-pyrene co-crystals were easily obtained by slow evaporation of the dichloromethane solution of H1 and pyrene (Fig. 1f). When yellow perylene and white H1 powder are added to chloroform, the solution turns green. High-quality dark green rectangular SOF-H1-perylene co-crystals were obtained by slowly diffusing *n*-hexane vapor into a chloroform solution of H1 and perylene (Fig. 1g). More interesting structural features emerge. As shown in Fig. 2, all three host–guest co-crystals exhibit 2D framework structures. The NDI moieties within the three planes of the triangular host macrocycle can interact with three guest molecules through face-to-face π–π interactions, resulting in the formation of a 2D structure in the *a*–*b* planes (Fig. 2b, e, and h). This process yields a perfectly hexagonal honeycomb porous framework, characterized by an equilateral hexagonal pore and six equilateral triangular pores in each layer (Fig. 2c, f, and i). The three NDI planes as electron acceptors can form face-to-face stacking through π–π interactions with the electron donor plane (DCA, pyrene, and perylene). The distances between the NDI moiety plane and the three donor planes are almost identical. The face-to-face π–π stacking distances between the NDI and the perylene, DCA, and pyrene are measured to be 3.32 Å, 3.38 Å, and 3.39 Å, respectively (Fig. 2b, e, and h).

**Fig. 3:**
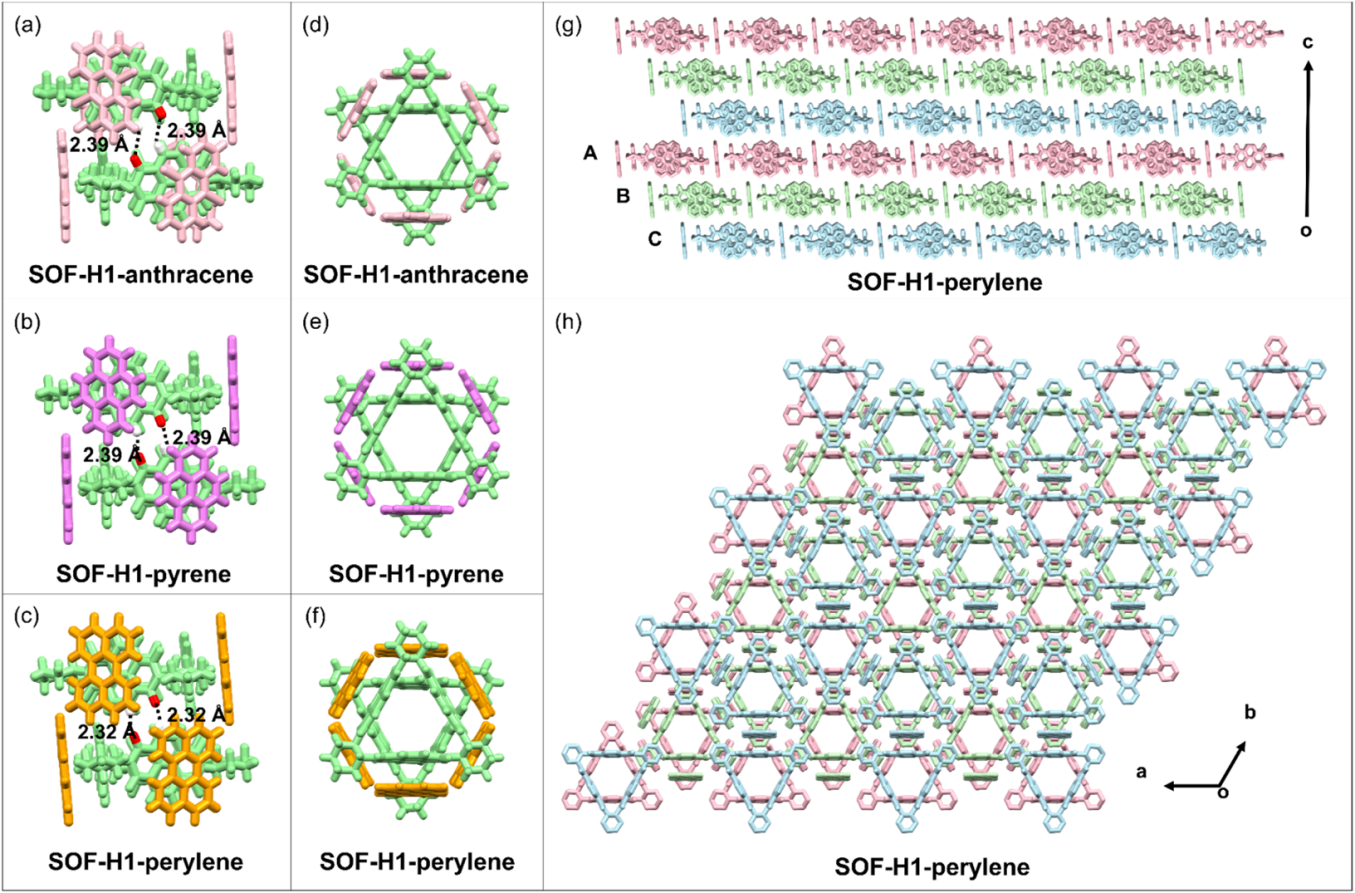
(a–c) The single-crystal structures of the three SOFs showing the H-bonding interactions between the adjacent layers. (d–f) The top view (along the *c*-axis) of the single-crystal structures of the adjacent two layers in the three SOFs. (g) The side view (along the *a*-axis) of the single-crystal structure of SOF-H1-pyrene as a representative example with two periodic ABC stackings (6 layers). (h) The top view (along the *a*-axis) of the single-crystal structure of SOF-H1-pyrene as a representative example with two periodic ABC stackings (6 layers). The *R* enantiomers were selected as representatives.

The hydrogen-bonding interactions along with the *c*-axis (between the 2D layers) stabilize the framework structure, endowing the material characteristics of SOFs. In the side view along the *a* or *b*-axis direction, the layered structures, which adopt ABC stacking modes, are clearly visible (Fig. 3). The self-assembly mode is identical in layers A, B, and C, differing only by a staggered shift (Fig. 3g and h). Along with the direction of the *c*-axis, multiple [C–H⋯O] hydrogen bonds between the H from the naphthalene and the O from the carbonyl connect each pair of triangular rings (Fig. 3a–c). The distances of the [C–H⋯O] hydrogen bonds are 2.32 Å, 2.39 Å, and 2.39 Å, respectively (Fig. 3a–c). To our surprise, the changes in the structures of the guest molecules do not affect the assembly pattern and the formation of the 2D framework structures (Fig. 3d–f). Interestingly, although the structures of the guest molecules have changed significantly, their cell parameters in the single crystals are almost unchanged. In the case of the *R*-SOF-H1-anthracene co-crystal, the structure corresponds to the *R*32 space group, with specific cell parameters as follows: *a* = 24.6999 Å, *b* = 24.6999 Å, *c* = 25.1460 Å, *α* = 90°, *β* = 90°, *γ* = 120° and volume = 13285.9 Å^3^. For the *R*-SOF-H1-pyrene co-crystal, the individual crystal structure conforms to the *R*32 space group, featuring specific cell parameters: *a* = 24.6162 Å, *b* = 24.6162 Å, *c* = 25.2652 Å, *α* = 90°, *β* = 90°, *γ* = 120° and volume = 13258.5 Å^3^. With regard to the *R*-SOF-H1-perylene co-crystal, the individual crystal structure also conforms to the *R*32 space group, featuring specific cell parameters: *a* = 24.0509 Å, *b* = 24.0509 Å, *c* = 25.9832 Å, *α* = 90°, *β* = 90°, *γ* = 120° and volume = 13016.3 Å^3^. The cell volumes of the SOF-H1-anthracene, SOF-H1-pyrene, and SOF-H1-perylene co-crystals are 13285.9 Å^3^, 13258.5 Å^3^, and 13016.3 Å^3^, respectively, showing a slight decreasing trend with increasing size of the guest molecules. The maximum variation in the volumes of the unit cell size is only 2%.

We also compared the unit cell parameters of the enantiomers. As expected, the *R* and *S* enantiomers of the three systems exhibited extremely similar parameters. The individual crystal structure of the *S*-SOF-H1-pyrene co-crystal accords with the *R*3 space group, featuring specific cell parameters of *a* = 24.6538 Å, *b* = 24.6538 Å, *c* = 25.2249 Å, *α* = 90°, *β* = 90°, *γ* = 120° and volume = 13277.9 Å^3^. Meanwhile, for the *S*-SOF-H1-anthracene co-crystal, the structure corresponds to the *R*32 space group, with specific cell parameters as follows: *a* = 24.6999 Å, *b* = 24.6999 Å, *c* = 25.1460 Å, *α* = 90°, *β* = 90°, *γ* = 120° and volume = 13285.9 Å^3^. As for the *S*-SOF-H1-perylene co-crystal, the individual crystal structure conforms to the *R*32 space group, featuring specific cell parameters as follows: *a* = 24.0466 Å, *b* = 24.0466 Å, *c* = 25.9712 Å, *α* = 90°, *β* = 90°, *γ* = 120° and volume = 13005.6 Å^3^.

Powder X-ray diffraction (PXRD) tests have been performed for all the co-crystals. As shown in Fig. S3,† the PXRD signals are rich and clear, and they align well with the simulated data, confirming the purity of the bulk materials. The thermogravimetric analysis (TGA) of all the SOF crystals has been performed. Similar tendencies were observed with two obvious steep drops for all three SOFs. The thermal stabilities of the three samples reach up to 250 °C (Fig. S4†).

This is really rare in the self-assembled single-crystal structure of the framework. Typically, slight adjustments in the structure of the guest molecules can cause significant changes in the entire single-crystal structure of the self-assembly. The appropriate sizes of the guest molecules and suitable D–A interactions between the host and guest are key to maintaining the 2D framework structure. The strong and suitable host–guest interactions in the plane of a single layer and the hydrogen-bonding interactions between the layers connect the host and guest molecules, finally forming the 2D framework structure.

**Fig. 4:**
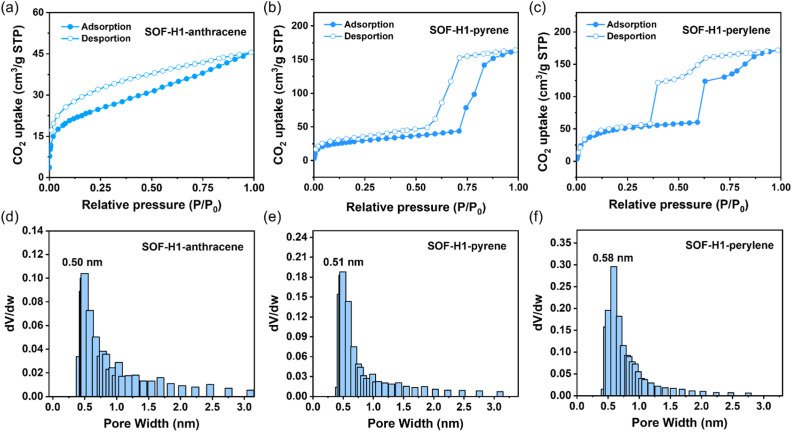
CO_2_ adsorption–desorption isotherms (195 K) of (a) SOF-H1-anthracene, (b) SOF-H1-pyrene, and (c) SOF-H1-perylene. Pore size distribution profiles of (d) SOF-H1-anthracene, (e) SOF-H1-pyrene, and (f) SOF-H1-perylene. The *R* enantiomers are selected as representatives.

Despite the staggered pattern mode adopted in the framework along the *c*-axis, the intrinsic pores are maintained due to the macrocyclic structure of the host molecules. We then studied the porous properties of the obtained SOF material. Before the gas adsorption tests, we first activated the three SOF crystals. The crystals were initially soaked in *n*-hexane for solvent exchange to remove the solvent molecules from the SOF crystals. The resulting samples were then further activated under a high vacuum at 50 °C for 12 hours to obtain desolvated crystals. The adsorption–desorption isotherms of this series of SOF materials were finally obtained by using CO_2_ molecules at 195 K (Fig. 4a–c). The CO_2_ isotherms show that the apparent Brunauer–Emmett–Teller (BET) surface areas of *R*-SOF-H1-anthracene, *R*-SOF-H1-pyrene, and *R*-SOF-H1-perylene are 90.6 m^2^ g^−1^, 106.7 m^2^ g^−1^, and 200.8 m^2^ g^−1^, respectively. The pore sizes of *R*-SOF-H1-anthracene, *R*-SOF-H1-pyrene, and *R*-SOF-H1-perylene were calculated using the Horvath–Kawazoe (HK) model, yielding values of 0.50 nm, 0.51 nm, and 0.58 nm, respectively (Fig. 4d–f). These results successfully demonstrated the porous nature of these SOF materials. Although the ABC stacking pattern occurs along the *c*-axis direction, the cavities of the host macrocyclic molecules remain interconnected. They are stacked in different layers by rotating 60° around the center of the triangle, forming a one-dimensional channel (Fig. 3h). The pores in the SOFs are originally induced by the chiral macrocyclic hosts themselves. The staggered stacking mode does not influence the formation of a through channel constructed by the host molecules themselves. Most importantly, due to the chiral cavities brought about by the host molecules, these channels possess chiral characteristics. The single-crystal structure of the macrocyclic host molecule itself has also been well investigated.^47,51,54,55^ In the absence of guest molecules, the self-assembly relying solely on the host molecules does not form a framework structure. It has been reported that the H1 molecule usually does not form a perfect ‘face-to-face’ stacking to finally realize the formation of 2D framework structures. The main weak interactions in the single-crystal host molecules are the π–π interactions between the NDI moieties. Either monoclinic *P*21 or triclinic *P*1 space groups, which are both non-chiral space groups, are obtained for the H1 single crystals under different crystallization conditions.

To achieve CPL, a crucial strategy involves inducing the aggregation of organic fluorophores into well-ordered assemblies.^56^ To date, a variety of CPL-active materials have been developed. However, due to the generally low dissymmetry factor (*g*_lum_) values of small organic molecules, multi-component assemblies are viewed as one of the most effective strategies for both CPL generation and the amplification of *g*_lum_.^57^ In order to enhance the photophysical and chiroptical properties, various compounds with extended π-conjugated systems have been introduced into chiral macrocycle solutions, aiming to facilitate the formation of ordered aggregates through D–A interactions. We thus used three guest molecules, DCA, pyrene, and perylene, to assemble with *R* and *S* triangular rings, resulting in six types of crystals (three pairs of enantiomers), achieving an ordered arrangement from disorder in the triangular ring assembly. As the structure of the guest molecules regularly changes, the corresponding shifts in the color and luminescent properties of the SOFs become visible to the naked eye. The host macrocyclic molecule H1 itself is a colorless crystal, while both SOF-H1-anthracene and SOF-H1-pyrene are red. The color of the SOF-H1-perylene crystal even becomes green. Thus, the photophysical properties of this series of SOF materials were subsequently studied.

**Fig. 5:**
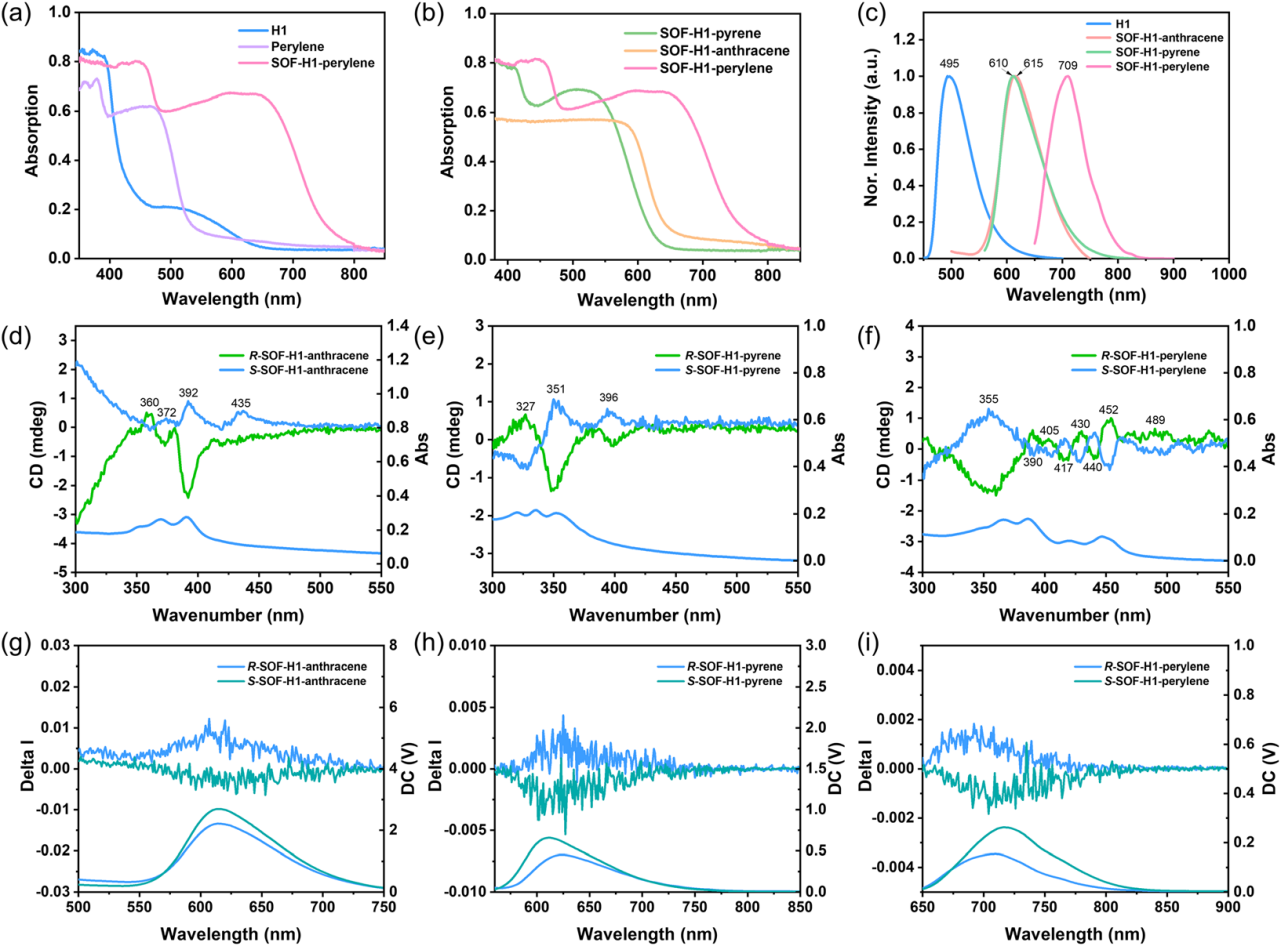
(a) The solid-state UV-Vis spectra (diffuse reflectance mode) of H1, perylene, and SOF-H1-perylene. (b) The solid-state UV-Vis spectra (diffuse reflectance mode) of SOF-H1-anthracene, SOF-H1-pyrene, and SOF-H1-perylene. (c) Normalized fluorescence spectra of SOF-H1-anthracene, SOF-H1-pyrene, and SOF-H1-perylene. (d–f) CD and UV-Vis spectra (transmission mode) of co-assemblies obtained by drop-casting from CH_2_Cl_2_. (g–i) CPL and PL spectra of *R*/*S*-SOF-H1-anthracene, *R*/*S*-SOF-H1-pyrene, and *R*/*S*-SOF-H1-perylene recorded at 298 K.

To elucidate the color changes of the co-crystals, the solid-state ultraviolet-visible (UV-vis) absorption spectra of SOF-H1-anthracene, SOF-H1-pyrene, SOF-H1-perylene, and their constituent monomers were recorded. Compared to the monomer H1, SOF-H1-perylene exhibits a significant red shift from ∼450 to ∼760 nm, which indicates strong charge transfer (CT) interactions and the formation of D–A complexes in the obtained frameworks (Fig. 5a). Similarly, strong CT interactions were also observed in the other two SOFs (Fig. S5†). As shown in Fig. 5b, SOF-H1-pyrene, SOF-H1-anthracene, and SOF-H1-perylene exhibit a gradual redshift trend with characteristic absorption edges at 630 nm, 659 nm, and 760 nm, respectively, which is attributed to the occurrence of n–π* or d–π* transitions. D–A interactions modify the molecular environment, leading to a decrease in the energy difference between the participating orbitals. The emission spectra also exhibit a similar trend of change. SOF-H1-anthracene, SOF-H1-pyrene, and SOF-H1-perylene exhibit emission peaks at 610 nm, 615 nm, and 709 nm, respectively. Notably, SOF-H1-perylene displays a pronounced redshift of ∼100 nm compared to SOF-H1-anthracene and SOF-H1-pyrene (Fig. 5c). This indicates that with an increase in the electron-donating ability of the donor molecules or an enlargement of the π plane, the emission spectrum progressively exhibits a redshift, accompanied by a reduction in the energy level spacing. The fluorescence lifetime of the compound is shown in Fig. S6–S8.† The absolute quantum yields of the three SOF crystals were also measured, which are 1.9% for *R*-SOF-H1-anthracene, 3.1% for *R*-SOF-H1-pyrene, and 2.1% for *R*-SOF-H1-perylene. It is evident that the optical properties of the luminescent materials can be tuned by manipulating the strong CT between the donor and acceptor.^58^

Chiral samples were prepared *via* the drop-casting method, followed by CD spectral measurements to confirm the transfer of chirality (Fig. S9†). As shown in Fig. S10,† the chiral macrocycles *R*-H1 and *S*-H1 exhibit good ground-state chirality in the thin film, with obvious Cotton effects at 333, 359, 368, and 389 nm, corresponding to the absorption peaks at 330, 349, 365, and 387 nm in the UV-Vis spectra. Further flipping of the sample also confirmed the reliability of the CD data (Fig. S11 and S12†). The CD signal peaks of *R*/*S*-SOF-H1-anthracene, *R*/*S*-SOF-H1-pyrene, and *R*/*S*-SOF-H1-perylene exhibited the Cotton effect at 360, 372, 392, and 435 nm; 327, 351, and 396 nm; 355, 390, 405, 417, 430, 440, 452, and 489 nm, respectively, showing significant changes, with a notable weakening of the Cotton effect, which basically corresponds to their UV-Vis absorption peaks (Fig. 5d–f). Compared with *R*/*S*-H1, the CD signals of the enantiomers of the three SOFs all show a significant red shift. The significant red shift of the CD signals relative to H1 demonstrates the successful transfer of chirality from H1 to the SOF skeleton. Taking the *R* configuration as an example, the negative Cotton effect near 400 nm is consistent with that of *R*-H1, clearly indicating that the chirality of the SOFs originates from the chiral macrocycle. To avoid the influence of linear dichroism (LD), we also conducted LD testing to ensure the authenticity of the CD (Fig. S13†). It is noteworthy that the original triangular macrocycle H1, despite exhibiting CD and weak fluorescence, lacked CPL (Fig. S10†). However, all three systems exhibited relatively CPL signals (Fig. 5g–i). The |*g*_lum_| values for SOF-H1-anthracene, SOF-H1-pyrene, and SOF-H1-perylene are 0.003, 0.002, and 0.006, respectively (Fig. S14†). The original data of the CPL tests from multiple angles can be found in Fig. S18–S25.†

Additionally, single-crystal structures reveal that upon the introduction of guest molecules, a strong charge transfer occurs between the electron-deficient macrocycle and the electron-rich guest. Facilitated by π–π stacking interactions, this charge transfer induces a reorganization of the molecular self-assembly within the co-crystal, ultimately resulting in the formation of a chiral helix (Fig. S15†). This also indicates that through supramolecular assembly, the chirality of cyclohexanediamine in the triangular ring has been transferred to the CT supramolecular assembly, achieving chirality transfer.^59^ This is the famous “sergeant-and-soldier” (SaS) principle, where a small number of chiral units (sergeants) can dictate the helical orientation of non-chiral units (soldiers) and induce the entire macromolecule to adopt a biased handedness. Chirality is transferred from the point chirality of cyclohexanediamine to 2D planar chirality fixed in a three-layer sandwich structure. The long-range order in the 2D SOFs further amplifies the chirality. The SaS effect has not only been extended to supramolecular polymer systems but has also been widely studied in liquid crystal aggregate systems.^60–62^

When chiral macrocyclic molecules tightly stack with achiral guest molecules through π–π interactions to form an ordered 2D co-assembled CT system, the chiral molecules influence the electron cloud distribution and orbital arrangement of the system, leading to the formation of chiral exciplexes. The close packing enhances the π–π interactions, leading to changes in the dipole moment. This chiral induction allows the overall system to exhibit chiral luminescence during the emission process, thereby generating circularly polarized light. Additionally, the geometric configuration of the SOF structure is crucial for the CPL performance. The formation of a 2D stacked structure through supramolecular self-assembly enables an ordered arrangement of molecular orbitals, which is more favorable for the transfer and amplification of chiral information, consequently enhancing the emission of CPL.

The differences in the |*g*_lum_| values among the three SOFs can be explained as follows: perylene possesses a larger π-plane than pyrene and DCA, which generally corresponds to higher electron density and enhanced conjugation effects. This enhancement facilitates stronger intermolecular interactions. The highest occupied molecular orbital (HOMO) energy level of perylene is higher than that of pyrene and DCA, leading to more pronounced π–π and CT interactions between perylene molecules and the macrocyclic components in the resulting co-crystals. Therefore, SOF-H1-perylene exhibits a higher |g_lum_| value compared to SOF-H1-pyrene and SOF-H1-anthracene.

**Fig. 6:**
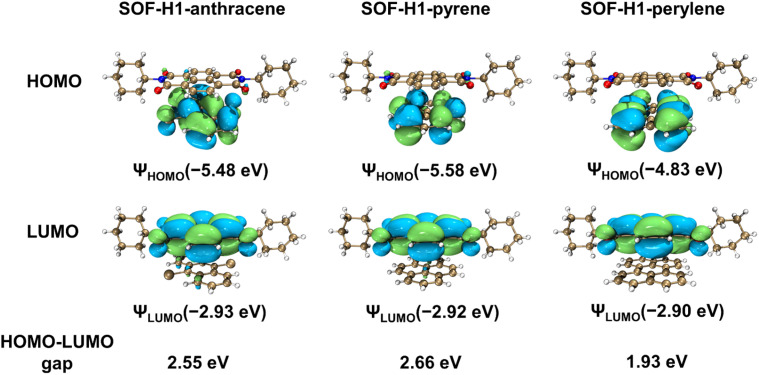
The HOMOs, LUMOs, and HOMO–LUMO energy gaps of the D–A fragments in SOF-H1-anthracene, SOF-H1-pyrene, and SOF-H1-perylene.

Density functional theory (DFT) calculations based on the solid superstructures of SOF-H1-anthracene, SOF-H1-pyrene, and SOF-H1-perylene were performed (Fig. 6). We employed the Gaussian 16 software package and the B3LYP method to calculate the HOMO–LUMO energy levels of the three different donor co-crystals, revealing their molecular orbital arrangements before and after cocrystallization, in order to understand the distinct photophysical properties of these three co-crystals.^63^ The HOMO levels of these three co-crystals are all located on the electron-donating molecules. Meanwhile, they exhibit similar lowest unoccupied molecular orbital (LUMO) energy levels, attributed to the concentration of their LUMO levels on the electron-accepting H1 (Fig. S26†). The HOMO–LUMO energy gaps of co-crystals SOF-H1-anthracene, SOF-H1-pyrene, and SOF-H1-perylene are calculated to be 2.55 eV, 2.66 eV, and 1.93 eV, and these values are consistent with their optical bandgaps. Molecules with large π planes can engage in π–π stacking interactions with the chiral molecule H1. This stacking effect modifies the electron cloud distribution and the charge transfer mechanism between the molecules, potentially inducing chirality in the excited state. These findings further reinforce our hypothesis.

## Conclusions

A series of chiral SOFs with similar 2D framework structures were constructed through host–guest interactions in the 2D plane and H-bonding interactions between the 2D layers. The change in the guest molecule structures did not affect the self-assembly pattern, and the 2D framework structures were maintained. Interestingly, the CPL activity can be tuned by modifying the structures of guest molecules. The incorporation of guest molecules structurally guarantees the formation of 2D frameworks, facilitates chirality transfer, and enhances the performance of CPL through strong CT mechanisms. The ability to precisely control the structure and functionality of the SOFs allows for the customization of their optical properties to match specific CPL requirements. This level of customization is crucial for developing advanced optical materials. Given the diversity and intrinsic characteristics of SOFs, the synergistic effects of their structure and host–guest interactions create a promising platform for designing advanced CPL-active composites, while also providing additional opportunities to enhance chiroptical properties. Overall, considering the limited number of CPL-active SOFs based on host–guest systems developed to date, there is significant potential for further advancements in this field.

## Author contributions

Y. Zhao conceived the idea and designed the experiments. J. Cui carried out the experiments. H. Wang performed the X-ray single crystal analysis. H. Liu directed the synthesis and single-crystal sample growth. H. Yu guided the calculation part. W. Wang guided the CPL applications. Y. Wang performed all the CPL tests. J. Cui and Y. Zhao co-wrote the manuscript. Y. Zhao directed the whole project.

## Conflicts of interest

There are no conflicts to declare.

## Supplementary Material

SC-016-D4SC08811E-s001

SC-016-D4SC08811E-s002
