# Supplementary material for: Single-crystal chiral two-dimensional supramolecular organic frameworks for tunable circularly polarized luminescence
Source: Chem Sci. 2025 Mar 19;16(17):7513–22. doi: 10.1039/d4sc08811e (PMC11951166; doi:10.1039/d4sc08811e)
Supplement: SC-016-D4SC08811E-s001 [file SC-016-D4SC08811E-s001.pdf]

Supplementary Information (SI) for Chemical Science.
This journal is © The Royal Society of Chemistry 2025

Supporting Information

Single-crystal chiral two-dimensional supramolecular organic frameworks for tunable circularly polarized luminescence

Jialin Cui,[a] Hui Wang,[a] Hui Liu,*[a] Hailong Yu,[a] Wei Wang,[b] Yu Wang,*[b] and Yingjie Zhao*[a]

[a] College of Polymer Science and Engineering, Qingdao University of Science and Technology, 53 Zhengzhou Road, 266000, Qingdao, China

[b] Shanghai Key Laboratory of Green Chemistry and Chemical Processes, School of Chemistry and Molecular Engineering, East China Normal University, 3663 N. Zhongshan Road, Shanghai 200062, China.

[*] Corresponding Authors' E-mail: hliu@qust.edu.cn; yuwang@chem.ecnu.edu.cn; yz@qust.edu.cn.

Table of Contents

1. Supporting Methods S2
   1.1 General materials and methods S2
   1.2 Synthesis procedure S3
   1.3 Crystal growth and characterization S4
   1.4 The single-crystal data of cocrystal S5
   1.5 Sample preparation for testing S7
   1.6 Theoretical calculations S7
2. Supporting Figures S8

1. Supporting Methods

1.1 General materials and methods

Unless otherwise indicated, all other reagents are commercially purchased and used without further purification. Organic solvents including methanol, chloroform, dichloromethane (CH_2Cl_2), N,N-dimethylformamide (DMF), and hexane were purchased from Tansoole. Naphthalene tetramethylanhydride, *(RR)*- and *(SS)-trans*-1,2-cyclohexanediamine, 9,10-dichloroanthracene (DCA), pyrene, and perylene were purchased from Energy Chemical. All aqueous solutions were prepared with Milli-Q water.

^{1}H and ^{13}C NMR spectra were performed on 400 MHz spectrometers (Bruker AVANCE NEO 400 Ascend) in the indicated solvents at room temperature. Chemical shifts were reported in δ (ppm) relative to TMS (δ = 0).

The solid-state UV-Vis absorbance was measured by UV spectrophotometer (Agilent, cary5000).

The transmitted UV-Vis absorbance was measured by a UV spectrometer (HITACHI, 3900).

Circular dichroism (CD) and Circularly Polarized Luminescence (CPL) spectra were measured on a Chirascan Series Spectrometer (Applied Photophysics Ltd, UK) at room temperature. Both the solution and solid-state CD tests were operated in transmittance mode.

Single-crystal data were measured using a Bruker D8 VENTURE X-ray single-crystal diffractometer and a Rigaku XtaLAB Synergy. The refinement and analysis were carried out using Olex2-1.3 software. The molecular stacking patterns were calculated using Mercury software (copyright by CCDC).

The fluorescence quantum yield was measured using the Quantaurus-QY (Hamamatsu Photonics) instrumen.

The crystal morphology was observed using a microscope (Nikon ECLIPSE LV100ND

POL, Japan).

1.2 Synthesis procedure

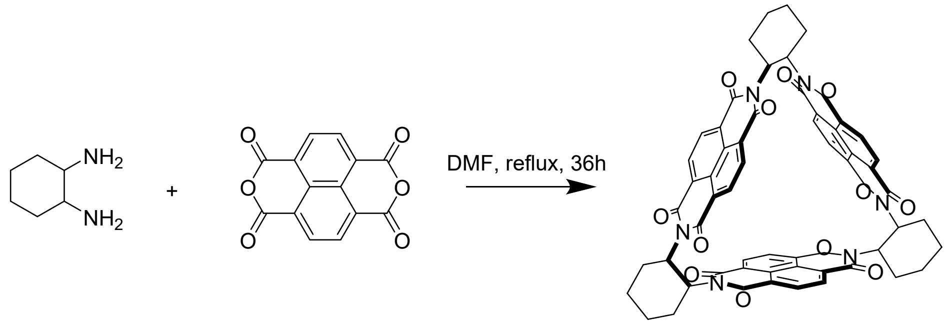

Scheme 1. Synthesis of compound ***R*** or ***S*-H1**.

***R*-H1**. Naphthalene tetracarboxylic dianhydride (12.3 g, 45.8 mmol) was dispersed in a 1 L single-neck flask equipped with a reflux condenser containing 450 mL of DMF. At room temperature, *(RR)-trans*-1,2-cyclohexanediamine (5.2 g, 45.8 mmol) was added to it. After the addition was complete, the mixture was heated to reflux at 150 °C for 36 hours. Upon completion of the reaction, the DMF solvent was removed by vacuum distillation. The resulting red solid was soaked in a dichloromethane/methanol solution (100:1, v/v) and sonicated for 10 minutes. The mixture was then filtered, and the filtrate was evaporated under reduced pressure. The product was further purified by column chromatography using dichloromethane as the eluent (200:1, v/v). The eluate was collected and concentrated to half its original volume. Subsequently, 10 times the volume of methanol was added to the concentrated solution, and the mixture was allowed to stand undisturbed overnight to precipitate a white solid. The white precipitate, identified as ***R*-H1** (3.2 g, 3.1 mmol), was collected with a yield of 20 %. This compound has been previously reported. ^{1}H NMR (400 MHz, $CDCl_3$) 8.49 (d, J = 1.5 Hz, 12H), 6.26 – 6.19 (m, 6H), 2.49 (d, J = 11.2 Hz, 6H), 2.03 – 1.91 (m, 14H), 1.75 – 1.61 (m, 6H).^{13}C NMR (100 MHz, $CDCl_3$): 162.78, 162.05, 131.38, 130.78, 126.48, 126.12, 125.86, 53.93, 29.96, 25.75.

***S*-H1**. The enantiomer ***S*-H1** (3.6 g, 3.5 mmol) was synthesized from naphthalene tetracarboxylic dianhydride (12.3 g, 45.8 mmol) and *(SS)-trans*-1,2-cyclohexanediamine (5.2 g, 45.8 mmol) following the same steps as ***R*-H1**, resulting in

a yield of 22%. The spectrum data of ***S*-H1** are identical to those of ***R*-H1**, except that its CD spectrum is the mirror image of ***R*-H1**.

1.3 Crystal growth and characterization

The SOFs with the same guest, either *R* or *S*, were prepared using the same method.

SOF-H1-pyrene. 8.0 mg of **H1** (0.008 mmol) and 6.0 mg of pyrene (0.030 mmol) are dissolved in 20 mL of CH_2Cl_2 to obtain a colorless, transparent solution. Upon evaporation of the CH_2Cl_2, dark red **SOF-H1-pyrene** crystals are formed at the bottom of the solution in one week.

SOF-H1-anthracene. 10.4 mg of **H1** (0.010 mmol) and 2.5 mg of 9, 10-dichloroanthracene (0.010 mmol) are dissolved in 10 mL of CH_2Cl_2, and the solution is filtered to remove any insoluble substances. High-quality red quadrangular **SOF-H1-anthracene** co-crystals are successfully obtained within one week by slowly diffusing methanol vapor into the filtrate.

SOF-H1-perylene. 12.5 mg of **H1** (0.012 mmol) and 9.2 mg of perylene (0.036 mmol) are dissolved in 20 mL of chloroform, and the solution is filtered to remove any insoluble substances. High-quality dark green square **SOF-H1-perylene** co-crystals are successfully obtained within three days by slowly diffusing n-hexane vapor into the filtrate.

1.4 The single-crystal data of cocrystal

Table S1. Crystal data and structure refinement.

CCDC	2391870	2063800	2391866
Identification code	**SOF-H1-anthracene *(R)***	**SOF-H1-pyrene *(R)***	**SOF-H1-perylene *(R)***
Empirical formula	$C_{55} H_{38} Cl_4 N_4 O_8$	$C_{57} H_{40} Cl_2 N_4 O_8$	$C_{33.5}H_{21}Cl_3N_2O_4$
Temperature/K	100	170	100
Space group	*R*32	*R*32	*R*32
a/Å	24.6956	24.6162	24.0509
b/Å	24.6956	24.6162	24.0509
c/Å	25.1324	25.2652	25.9832
α/°	90	90	90
β/°	90	90	90
γ/°	120	120	120
Volume/Å^3	13274.1	13258.5	13016.3
Z	9	3	18
Data completeness	1.000	0.998	1.000
R(reflections)	0.1289	0.0646	0.1999
wR2(reflections)	0.3274	0.0587	0.1955

CCDC	2391868	2391869	2391866
Identification code	**SOF-H1-anthracene *(S)***	**SOF-H1-pyrene *(S)***	**SOF-H1-perylene *(S)***
Empirical formula	$C_{55} H_{38} Cl_4 N_4 O_8$	$C_{57} H_{40} Cl_2 N_4 O_8$	$C_{30}H_{20}N_3O_7$
Temperature/K	100	170	100
Space group	*R*32	*R*3	*R*32
a/Å	24.7120	24.6536	24.0466
b/Å	24.7120	24.6536	24.0466
c/Å	25.1045	25.2258	25.9712
α/°	90	90	90
β/°	90	90	90
γ/°	120	120	120
Volume/Å^3	13276.9	13278.1	13005.6
Z	9	9	18
Data completeness	1.000	1.000	1.000
R(reflections)	0.0749	0.1459	0.2458
wR2(reflections)	0.2266	0.1310	0.2066

1.5 Sample preparation for testing

The self-assembly process in dichloromethane. The ***R*-** and (or ***S*-H1**) powder (2.0 mg, 1.936 μmol) was dissolved in 1.0 mL of dichloromethane. A small amount of insoluble solid was filtered and removed using a 0.45 μm microporous membrane to form a clear and transparent solution (1.9 mM). The self-assembly of ***R/S*-H1** was prepared by drop-casting the corresponding dichloromethane solution onto a quartz wafer.

Other compounds used for CD and CPL tests, as well as the UV–vis absorption tests of the compound films, were also prepared by drop-casting solutions with a concentration of approximately 2.0 mM using the same method.

1.6 Theoretical calculations

The intermolecular potentials were calculated using the UNI force field by Mercury software (copyright CCDC). Moreover, the calculations of the energy levels were using density functional theory (DFT) by Gaussian 16 package. The structure of the D-A molecular pair was directly imported from the crystal document and the molecular orbitals were obtained at the B3LYP/6-31G (d,p) level. The electron excitation analysis was carried out by Multiwfn.

2. Supporting Figures

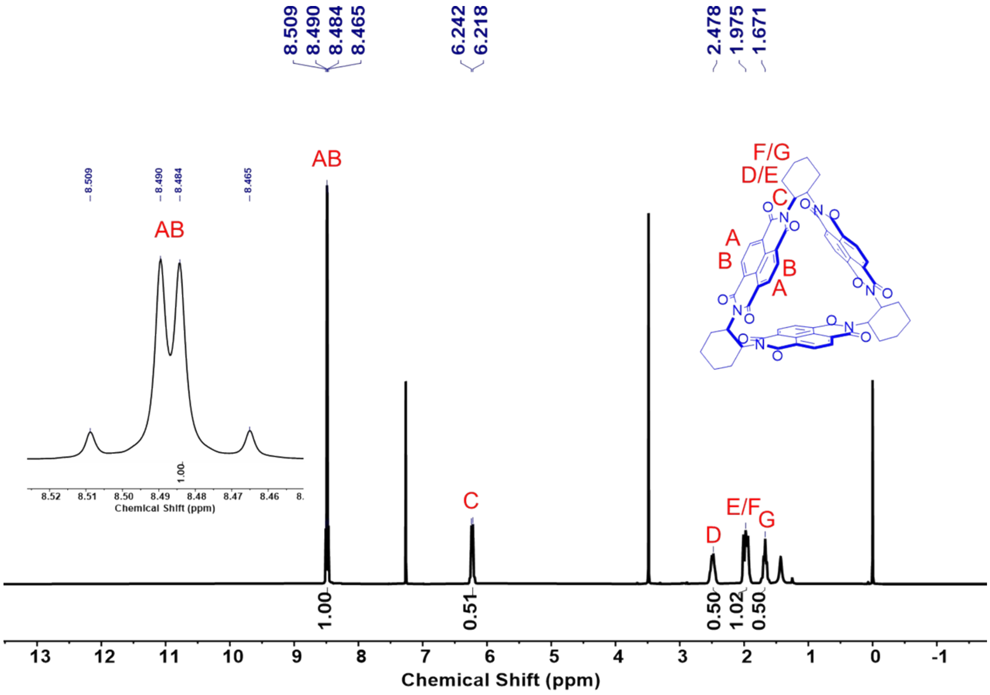

Figure S1. ^{1}H NMR spectrum (400 MHz, 298 K, $CDCl_3$) of **H1**.

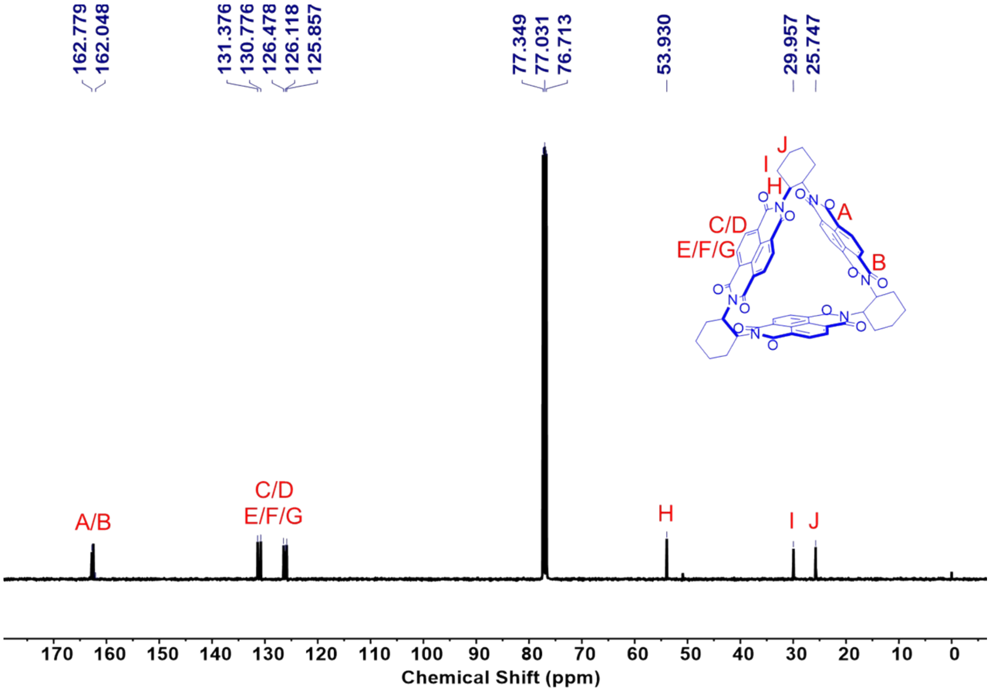

Figure S2. ^{13}C NMR spectrum (400 MHz, 298 K, $CDCl_3$) of **H1**.

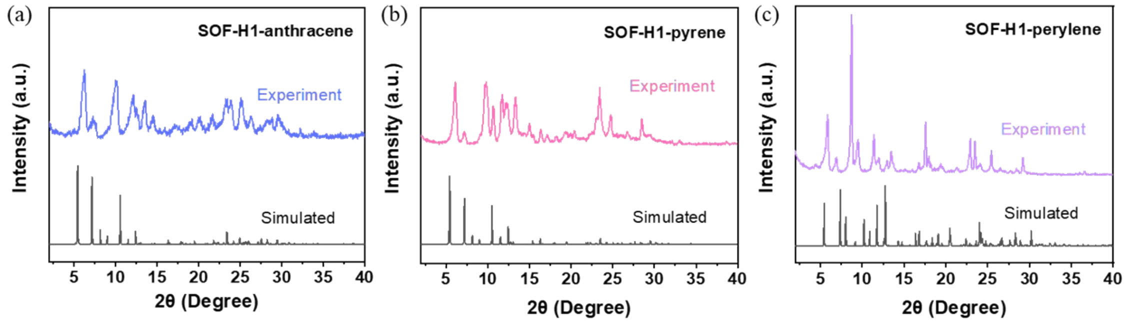

Figure S3. Powder X-ray Diffraction (PXRD) spectra of (a) **SOF-H1-anthracene**, (b) **SOF-H1-pyrene**, and (c) **SOF-H1-perylene**.

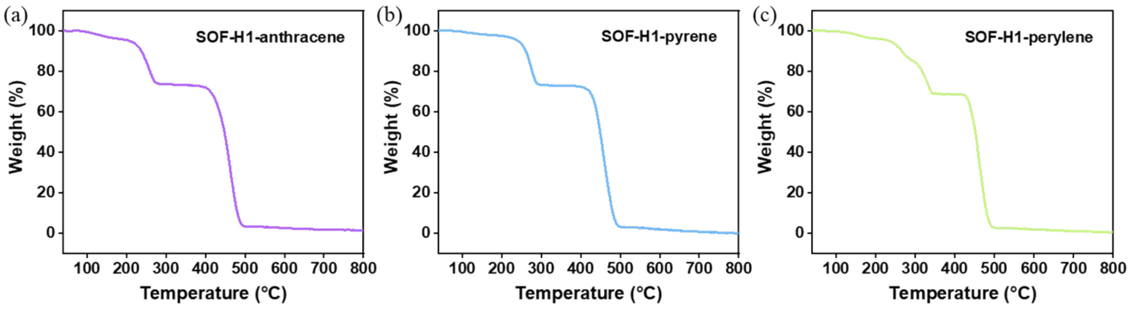

Figure S4. Thermogravimetric Analysis (TGA) spectra of (a) **SOF-H1-anthracene**, (b) **SOF-H1-pyrene**, and (c) **SOF-H1-perylene**.

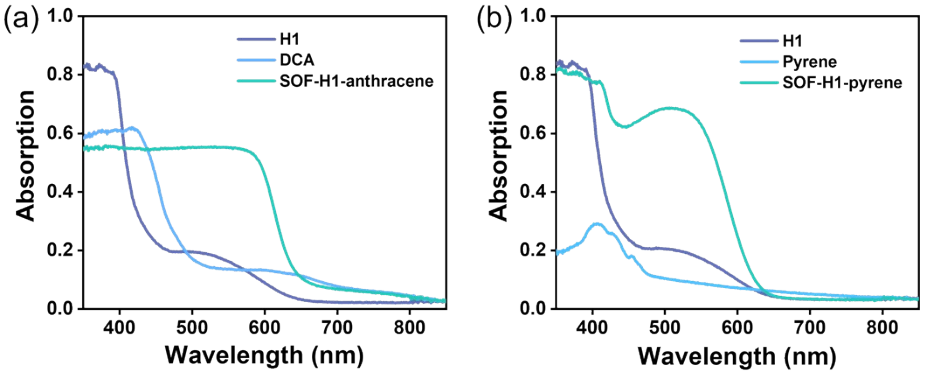

Figure S5. (a) The solid-state ultraviolet-visible (UV-vis) absorption spectra of **H1**, **DCA**, and **SOF-H1-anthracene**. (b) The UV-vis absorption spectra of **H1**, Pyrene, and **SOF-H1-pyrene**.

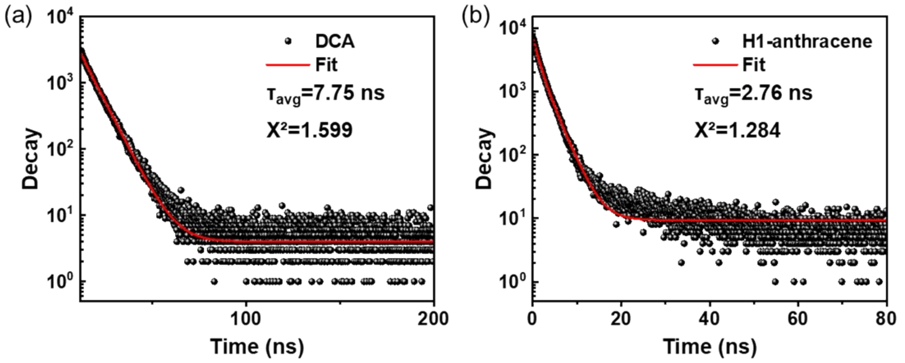

Figure S6. The fluorescence decay curves and lifetime for (a) the guest DCA, and (b) the co-crystal ***R*-SOF-H1-anthracene**. λ_{ex} = 405 nm.

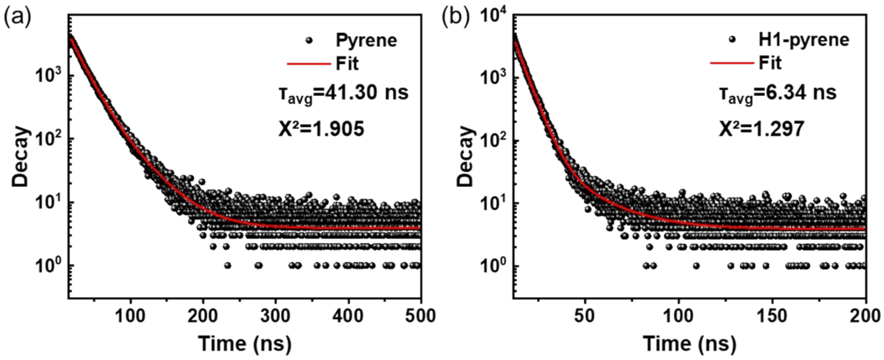

Figure S7. The fluorescence decay curves and lifetime for (a) the guest Pyrene, and (b) the co-crystal ***R*-SOF-H1-pyrene**. λ_{ex} = 405 nm.

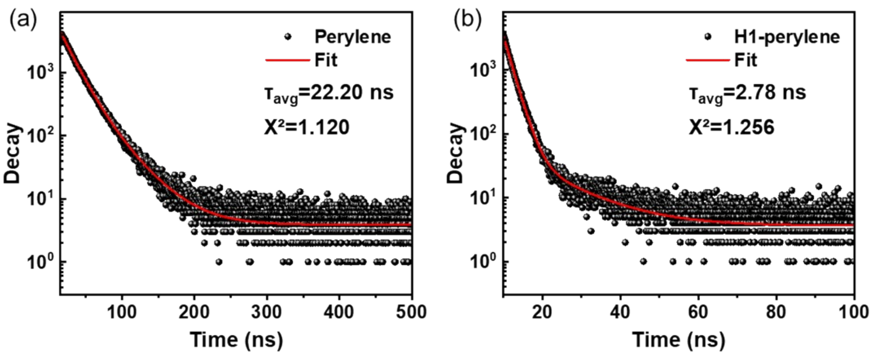

Figure S8. The fluorescence decay curves and lifetime for (a) the guest Perylene, and (b) the co-crystal ***R*-SOF-H1-perylene**. λ_{ex} = 405 nm.

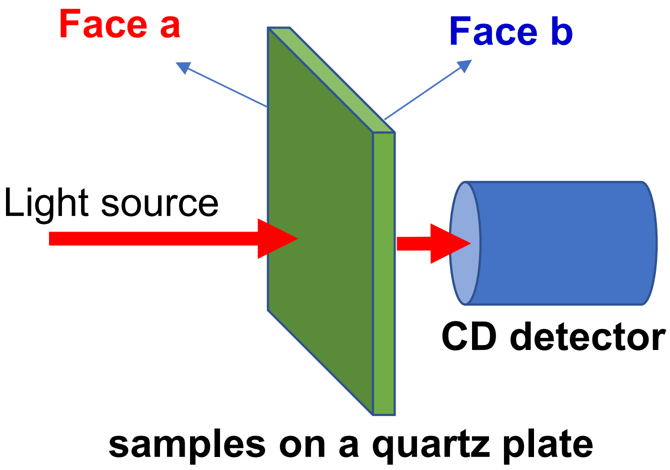

Figure S9. A diagram of the set up for measuring the CD of drop-casting film (side view).

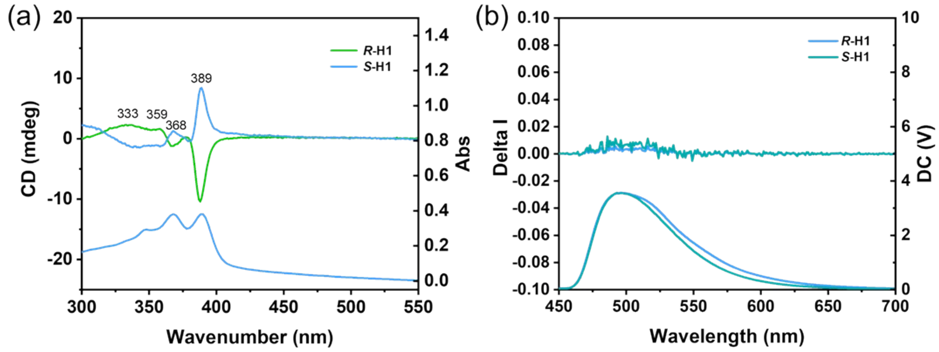

Figure S10. (a) CD and UV spectra of of ***R/S*-H1** self-assemblies obtained by drop-casting from CH_2Cl_2. (b) CPL and DC spectra of ***R/S*-H1**.

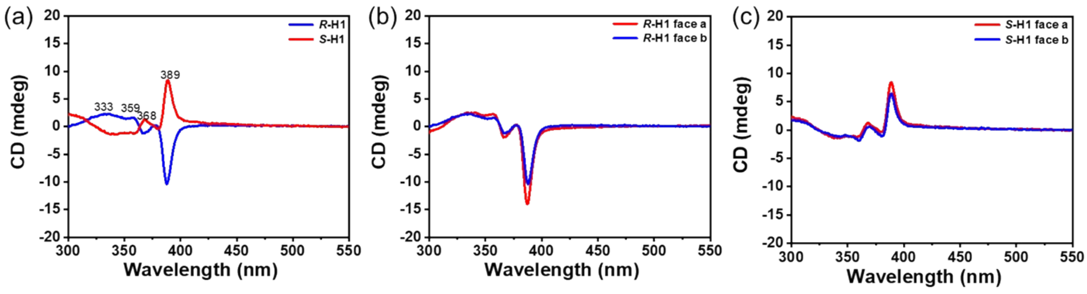

Figure S11. Solid-state CD spectra recorded at 298 K of (a) ***R/S*-H1**, (b) ***R*-H1** in different orientations, and (c) ***S*-H1** in different orientations.

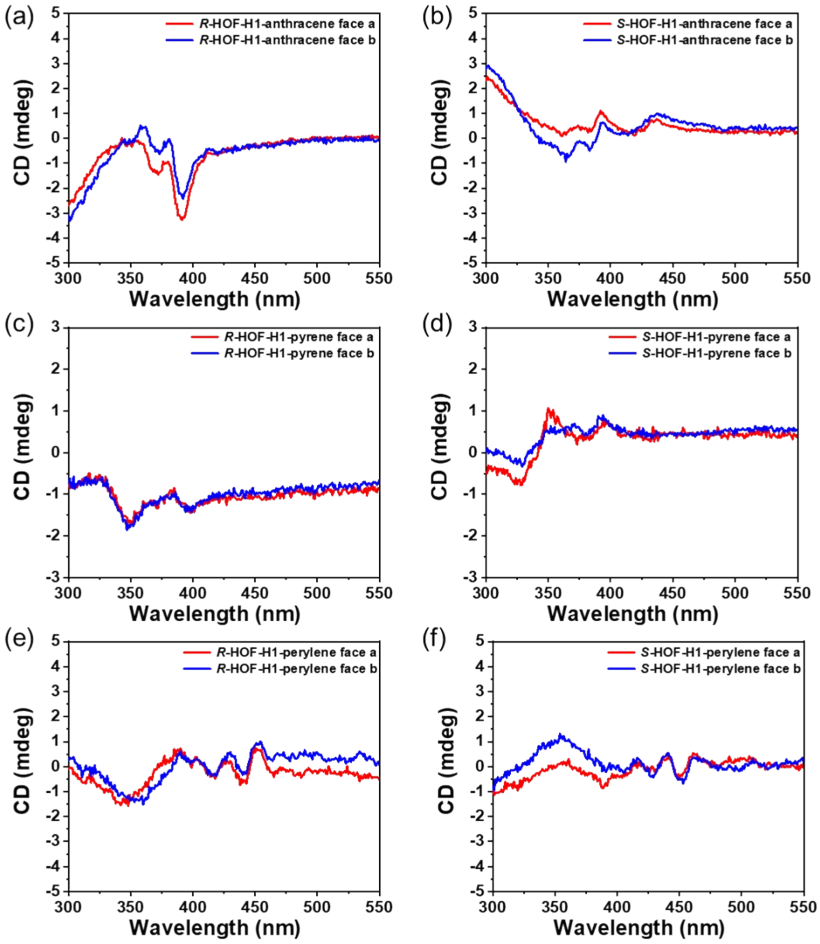

Figure S12. Solid-state CD spectra recorded at 298 K of (a-b) ***R/S*-SOF-H1-anthracene**, (c-d) ***R/S*-SOF-H1-pyrene**, and (e-f) ***R/S*-SOF-H1-perylene** in different orientations.

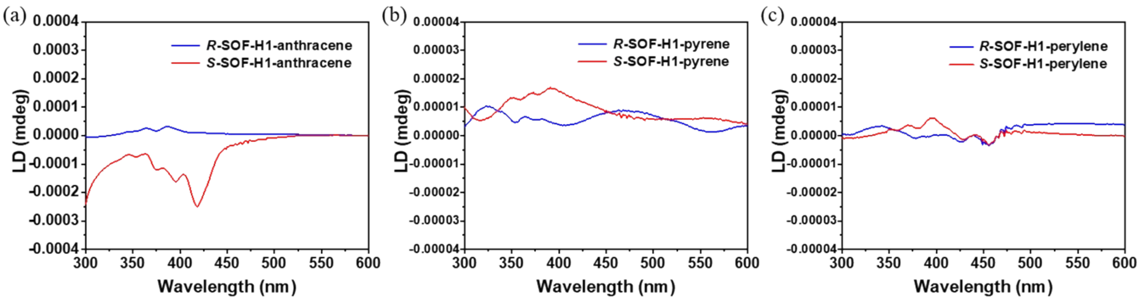

Figure S13. LD spectra of (a) ***R/S*-SOF-H1-anthracene**, (b) ***R/S*-SOF-H1-pyrene**, (c) ***R/S*-SOF-H1-perylene** recorded at 298 K.

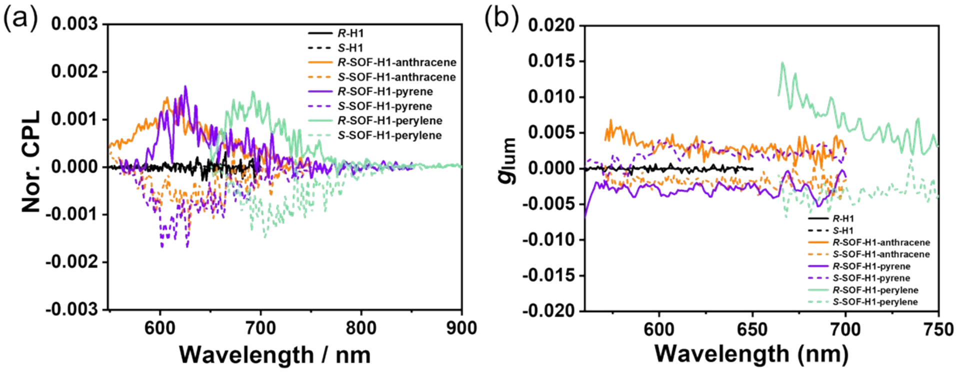

Figure S14. Nor. CPL and g_{lum} of ***R/S*-SOF-H1-anthracene**, ***R/S*-SOF-H1-pyrene**, ***R/S*-SOF-H1-perylene**, and **H1** recorded at 298 K.

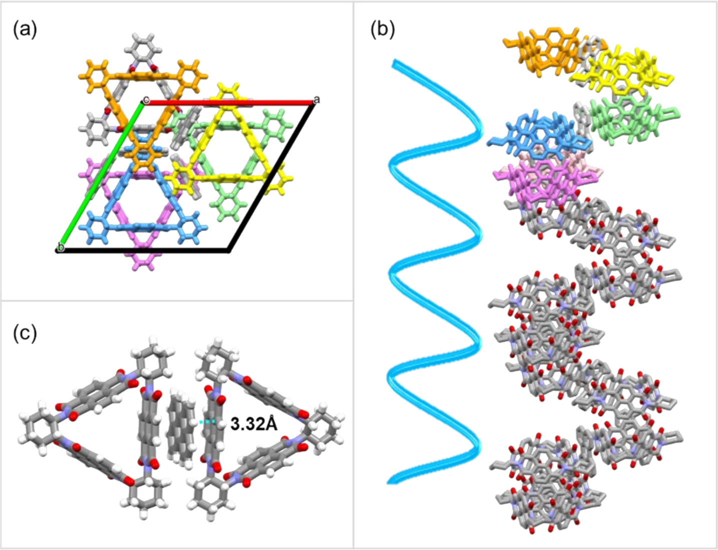

Figure S15. The top view (a) and a front view (b) of a self-assembled helix in the crystal structure of ***R*-SOF-H1-perylene**. (c) A schematic diagram of the π-π interaction between the electron-deficient NDI plane of **H1** and the electron-rich perylene guest.

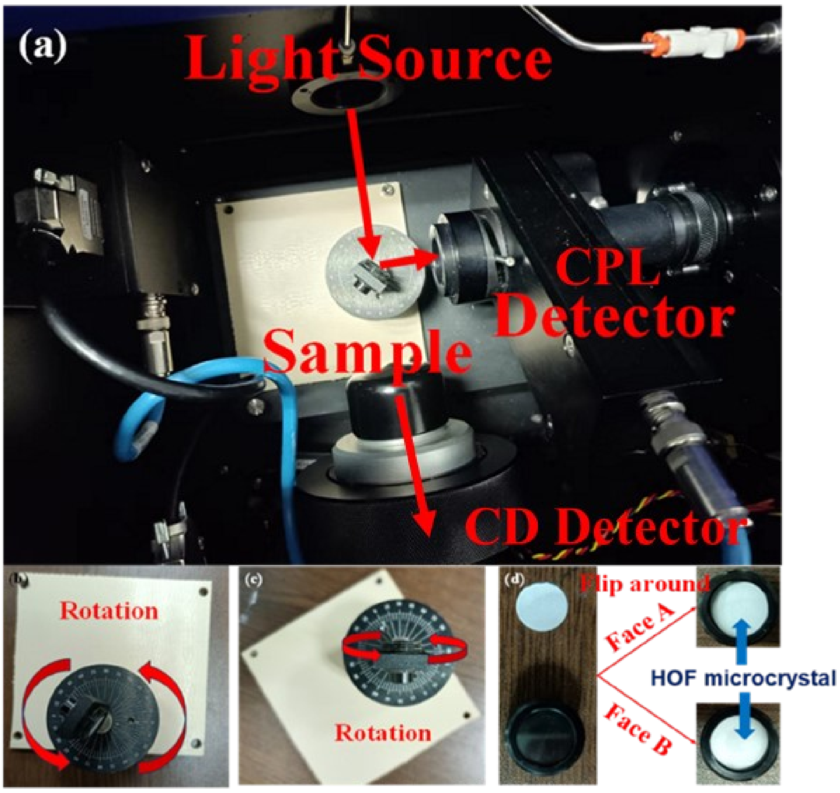

Figure S16. (a) CD and CPL test setup of Chirascan Series Spectrometer (Applied Photophysics Ltd, UK); (b, c) The rotation method of the samples for CPL tests; (d) The flip around method of the samples for CPL tests.

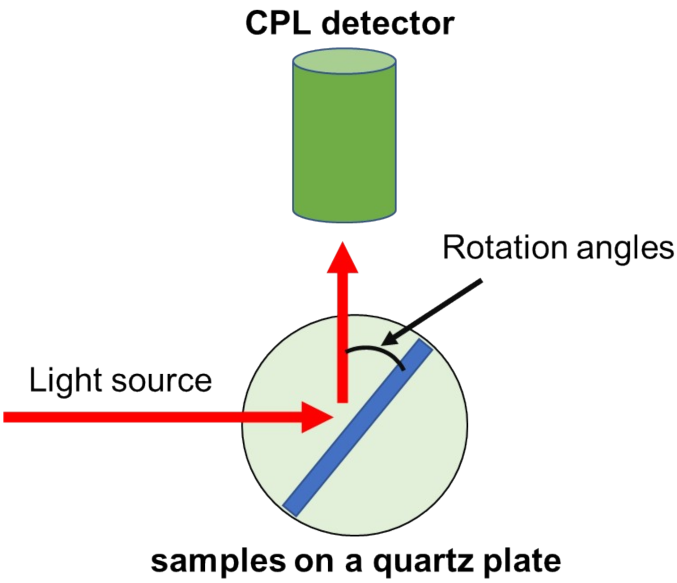

Figure S17. A diagram of the set up for measuring the CPL of drop-casting film (top view).

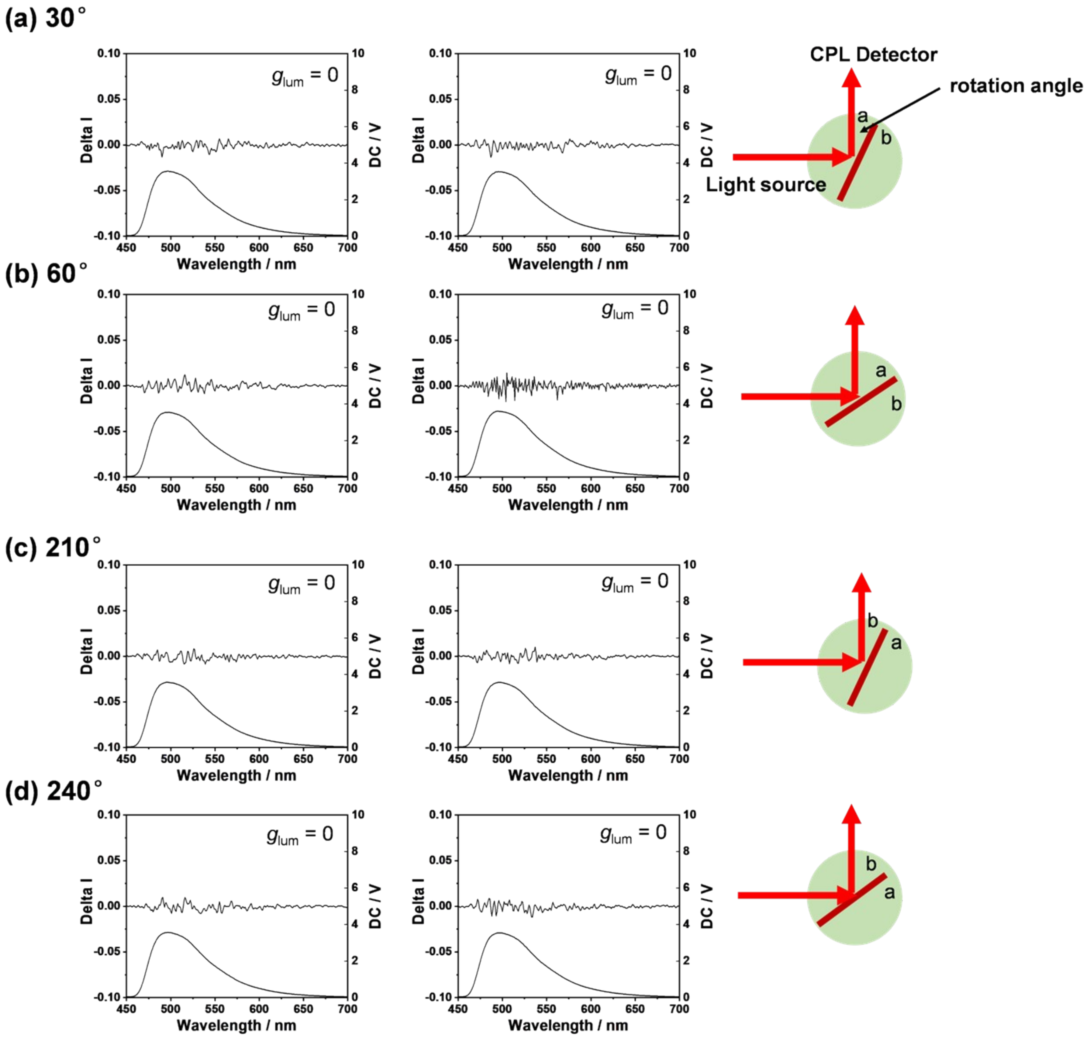

Figure S18. The CPL, DC spectra and corresponding g_{lum} of ***R*-H1** in film state measured at rotation angles of (a) 30º, (b) 60º, (c) 210º, and (d) 240º. Each angle was remeasured 2 times to eliminate systematic and random error.

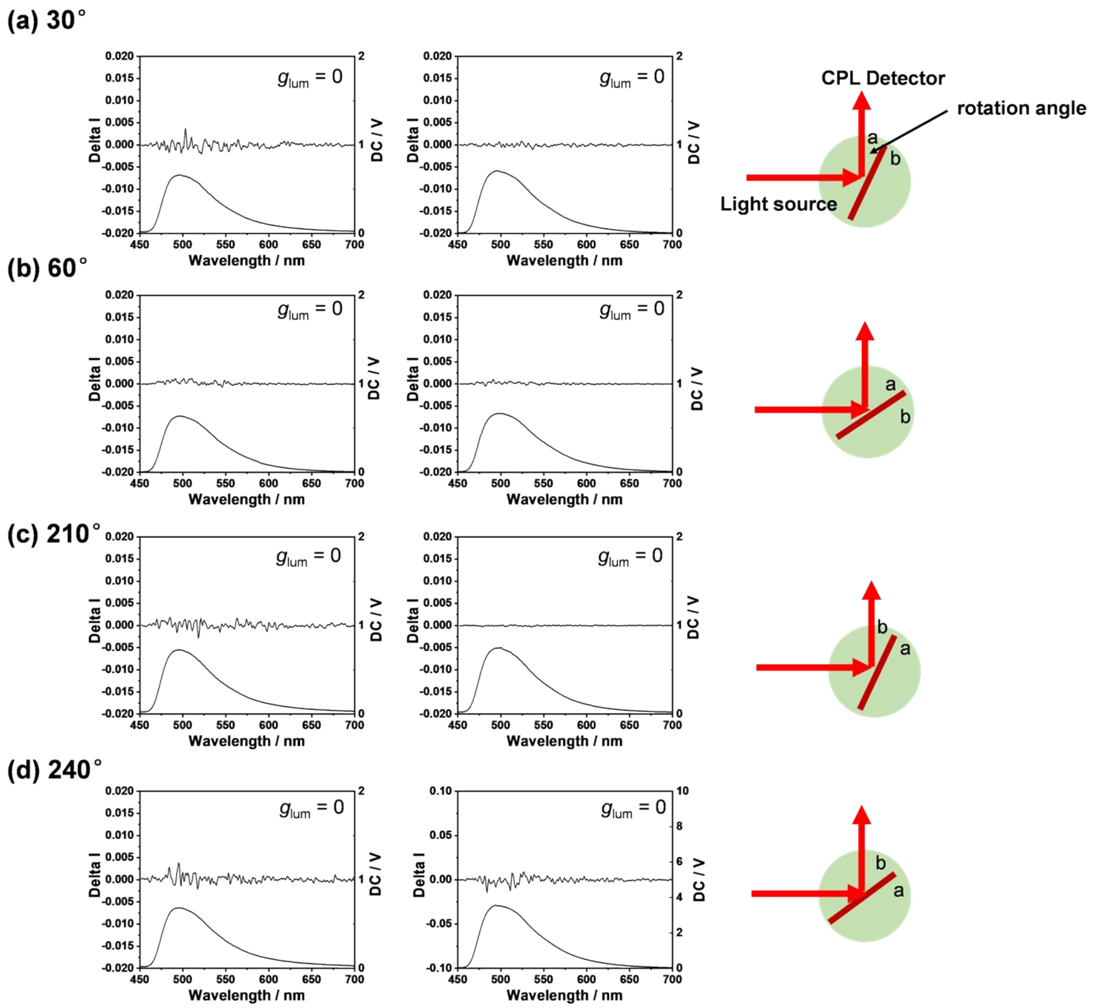

Figure S19. The CPL, DC spectra and corresponding g_{lum} of ***S*-H1** in film state measured at rotation angles of (a) 30º, (b) 60º, (c) 210º, and (d) 240º. Each angle was remeasured 2 times to eliminate systematic and random error.

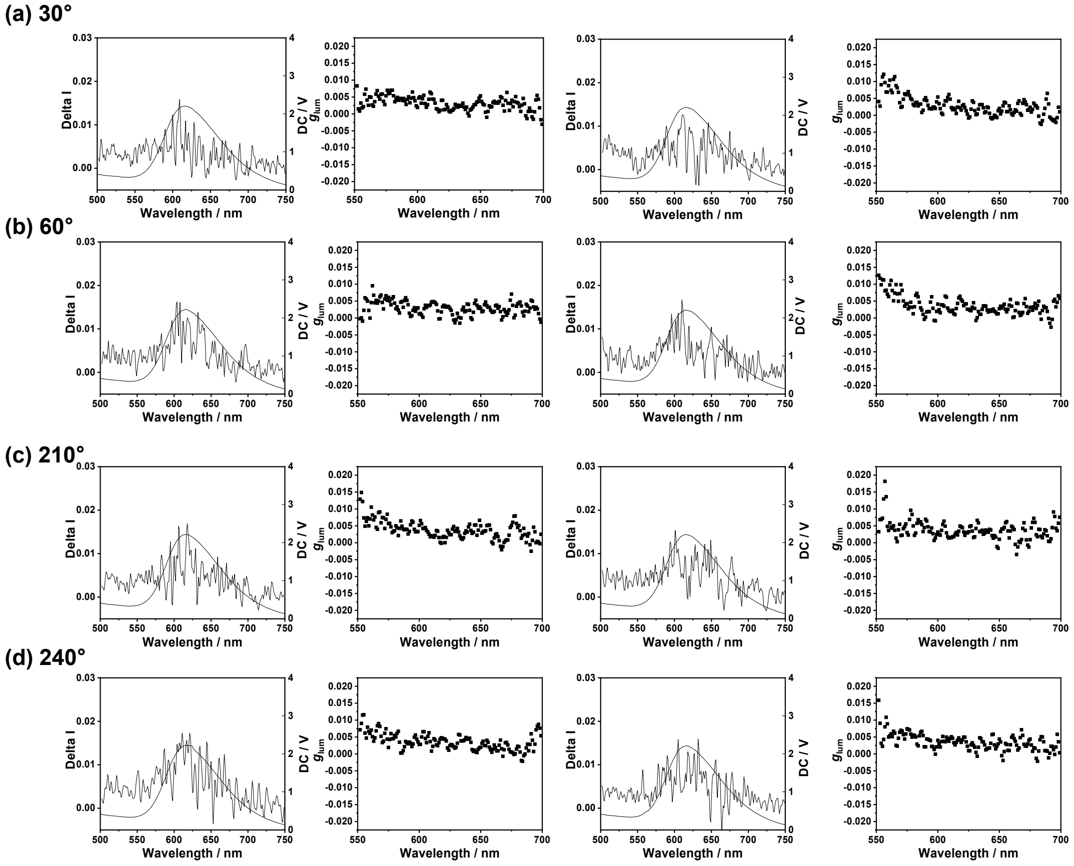

Figure S20. The CPL, DC spectra and corresponding g_{lum} of ***R*-HOF-H1-anthracene** in film state measured at rotation angles of (a) 30º, (b) 60º, (c) 210º, and (d) 240º. Each angle was remeasured 2 times to eliminate systematic and random error.

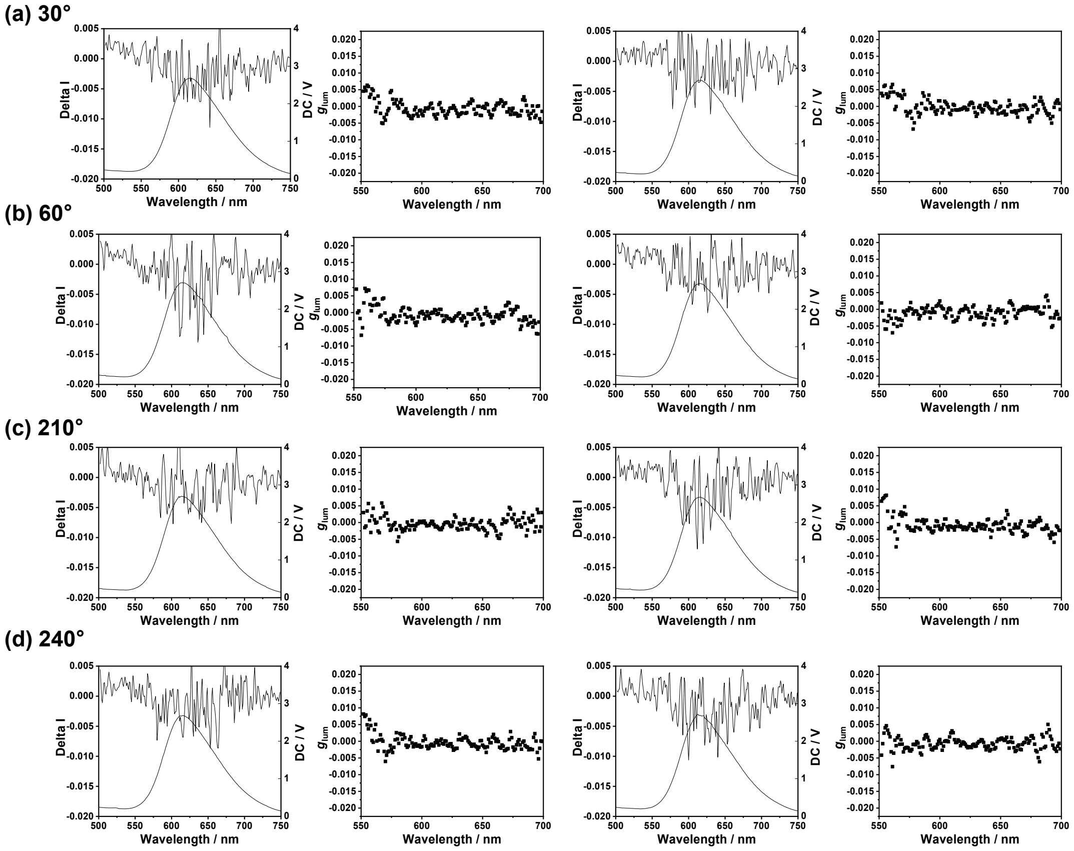

Figure S21. The CPL, DC spectra and corresponding g_{lum} of ***S*-HOF-H1-anthracene** in film state measured at rotation angles of (a) 30º, (b) 60º, (c) 210º, and (d) 240º. Each angle was remeasured 2 times to eliminate systematic and random error.

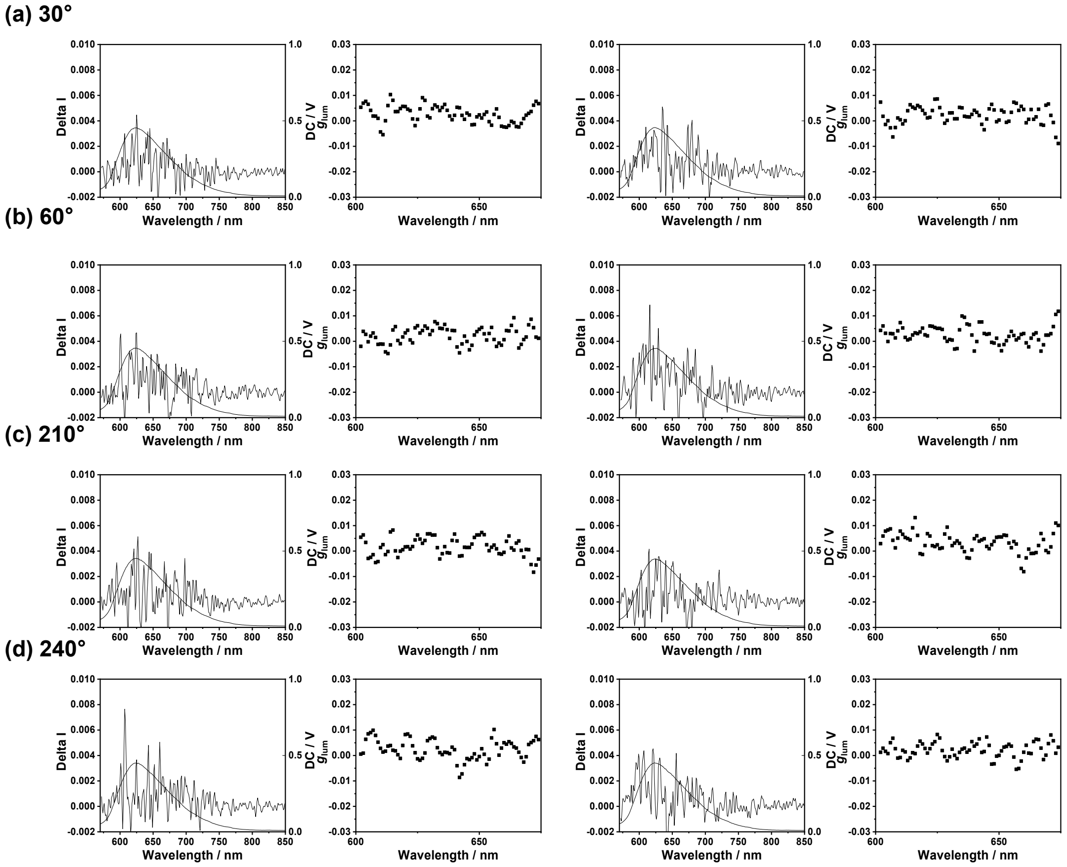

Figure S22. The CPL, DC spectra and corresponding g_{lum} of ***R*-HOF-H1-pyrene** in film state measured at rotation angles of (a) 30º, (b) 60º, (c) 210º, and (d) 240º. Each angle was remeasured 2 times to eliminate systematic and random error.

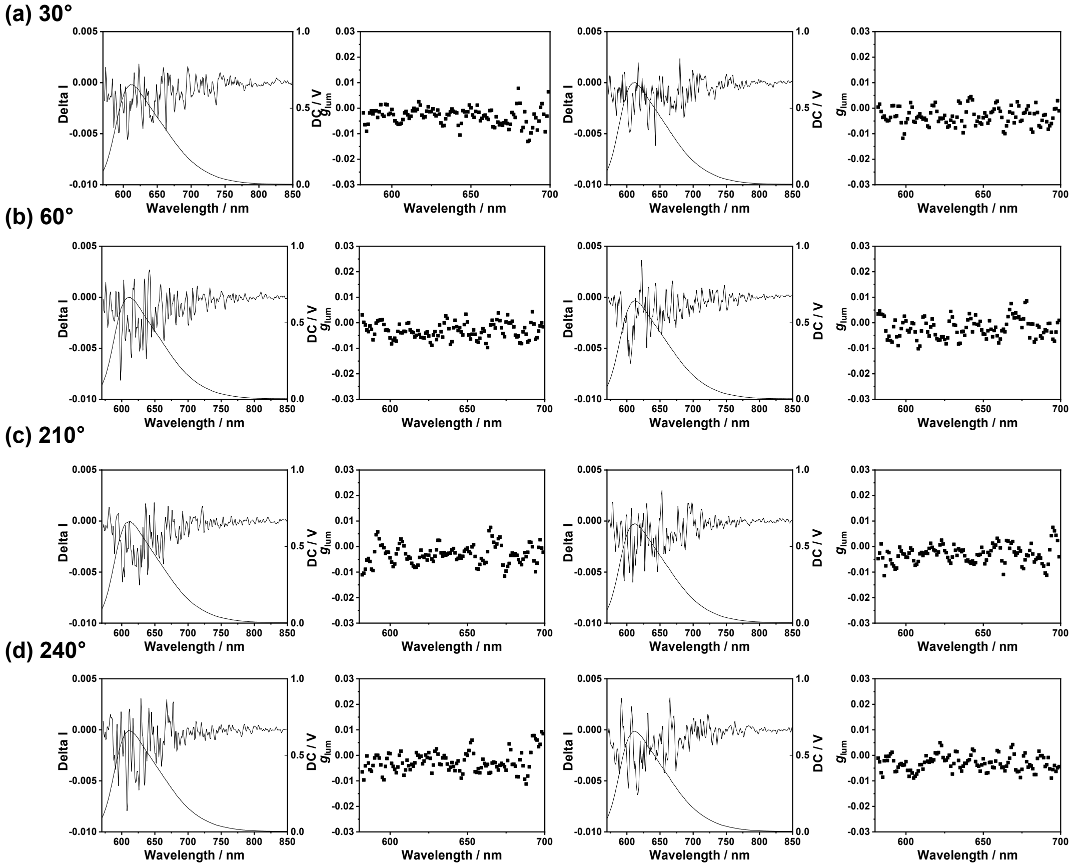

Figure S23. The CPL, DC spectra and corresponding g_{lum} of ***S*-HOF-H1-pyrene** in film state measured at rotation angles of (a) 30º, (b) 60º, (c) 210º, and (d) 240º. Each angle was remeasured 2 times to eliminate systematic and random error.

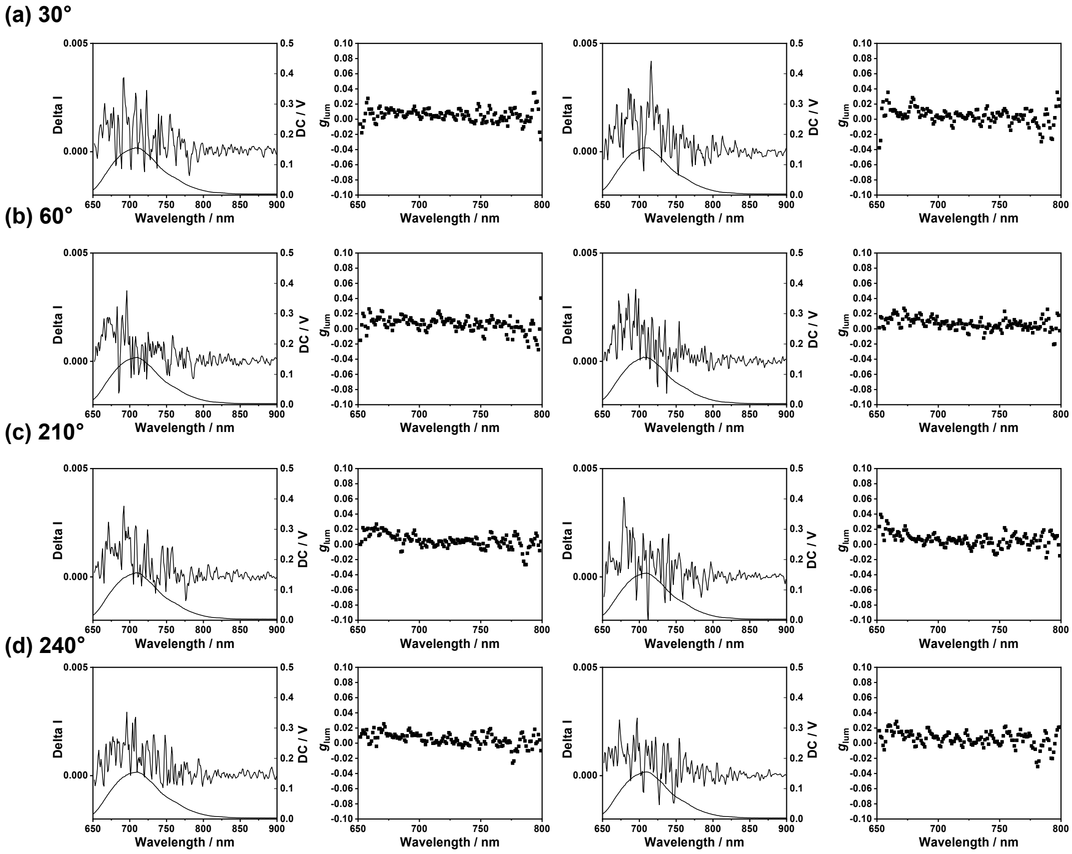

Figure S24. The CPL, DC spectra and corresponding g_{lum} of ***R*-HOF-H1-perylene** in film state measured at rotation angles of (a) 30º, (b) 60º, (c) 210º, and (d) 240º. Each angle was remeasured 2 times to eliminate systematic and random error.

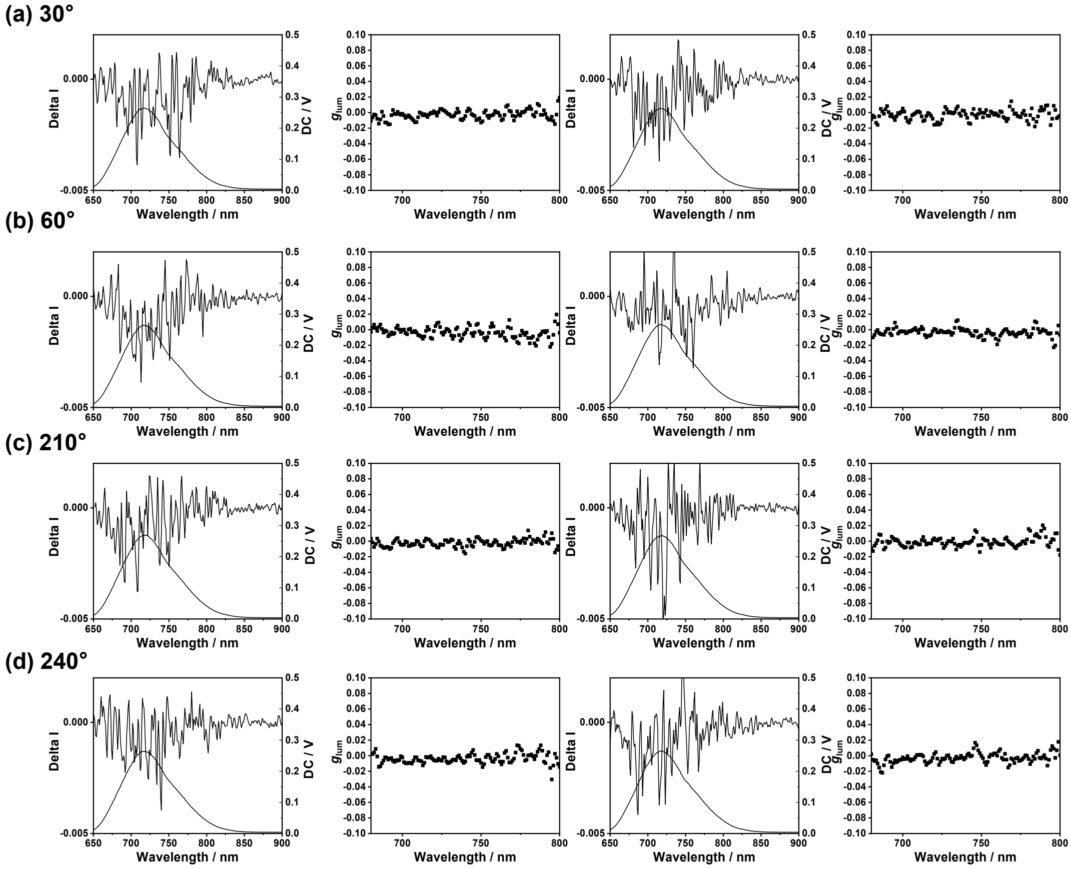

Figure S25. The CPL, DC spectra and corresponding g_{lum} of ***S*-HOF-H1-perylene** in film state measured at rotation angles of (a) 30º, (b) 60º, (c) 210º, and (d) 240º. Each angle was remeasured 2 times to eliminate systematic and random error.

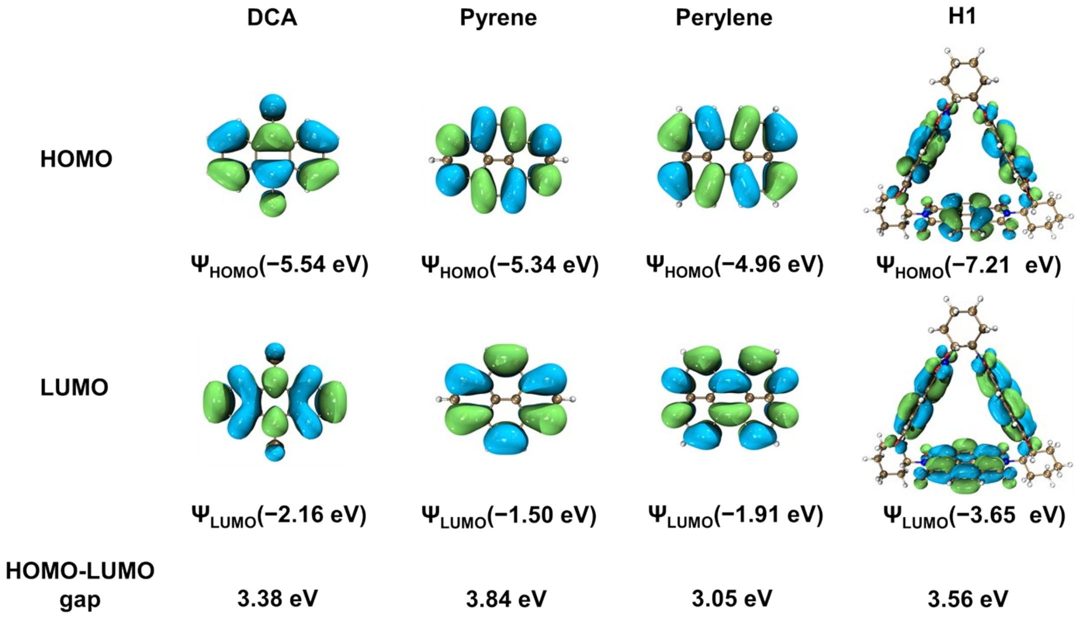

Figure S26. The HOMOs, LUMOs and HOMO-LUMO energy gaps of **DCA**, pyrene, perylene, and **H1** obtained through DFT calculations.
